# Supplementary material for: Hijacking a bacterial ABC transporter for genetic code expansion
Source: Nature. 2025 Oct 15;647(8091):1045–53. doi: 10.1038/s41586-025-09576-w (PMC12657241; doi:10.1038/s41586-025-09576-w)
Supplement: Supplementary file 1 — This file contains Supplementary Figs. 1–21, Supplementary Methods, Supplementary Tables 1–7 and protein sequences. [file 41586_2025_9576_MOESM1_ESM.pdf]

nature portfolio

https://doi.org/10.1038/s41586-025-09576-w

Supplementary information

Hijacking a bacterial ABC transporter for genetic code expansion

In the format provided by the authors and unedited

Supplementary Information for:

Hijacking a bacterial ABC transporter for genetic code expansion

Tarun Iype,[1]# Maximilian Fottner,[1]# Paul Böhm,[1] Carlos Piedrafita,[2] Yannis Möller[1], Michael Groll[2] and Kathrin Lang[1,2]*

[1]Department of Chemistry and Applied Biosciences (D-CHAB), ETH Zurich, Vladimir-Prelog-Weg 3, 8093 Zurich, Switzerland

[2]Technical University of Munich, TUM School of Natural Sciences, Department of Bioscience, Center for Protein Assemblies, 85748 Garching, Germany

# these authors contributed equally to this work

* to whom correspondence should be addressed: kathrin.lang@org.chem.ethz.ch

Table of Contents

Supplementary Figures 1-21 3
General methods: Plasmids and Reagents 32
Synthesis of peptides via solid phase peptide synthesis 33
Scheme S1: Fmoc SPPS strategy used for synthesis of peptides in this study. 33
Expression and purification of OppA and variants 34
Crystallization and structure determination of the OppA:G-SisoK complex 34
Expression and purification of Tyrosinase 35
Expression and purification of eGFP nanobodies and their yield determination 35
Evolution of HisoKRS 35
On bead CuAAC labeling of eGFP-NB with Picolyl-Azide-Sulfo-Cy5 36
Photocrosslinking of diazirine bearing proteins in cells 36
Tyrosinase-mediated labelling of PisoK bearing proteins 36
Chemical crosslinking of protein-protein complexes using ClAisoK in living E. coli 37
Affibody-ProteinZ: 37
sfGFP dimer: 37
Rab1b-DrrA 37
Chemical crosslinking of Affibody and ProteinZ in vitro 37
Determination of doubling times of isoK12 and K12 in AI and 2-YT media 37
Platereader based sfGFP fluorescence measurements 38
Table 1: Plasmids used in this study 38
Table 2: Primers used in this study 48
Table 3: Primers used for SLIM cloning 49
Table 4: Peptides used in this study 49
Table 5 Crystallographic data collection and refinement statistics. 51
Table 6: Mutations in OppA variants 52
Table 7: gRNA sequences used in this study: 52
Protein Sequences 53

Supplementary Figures 1-21

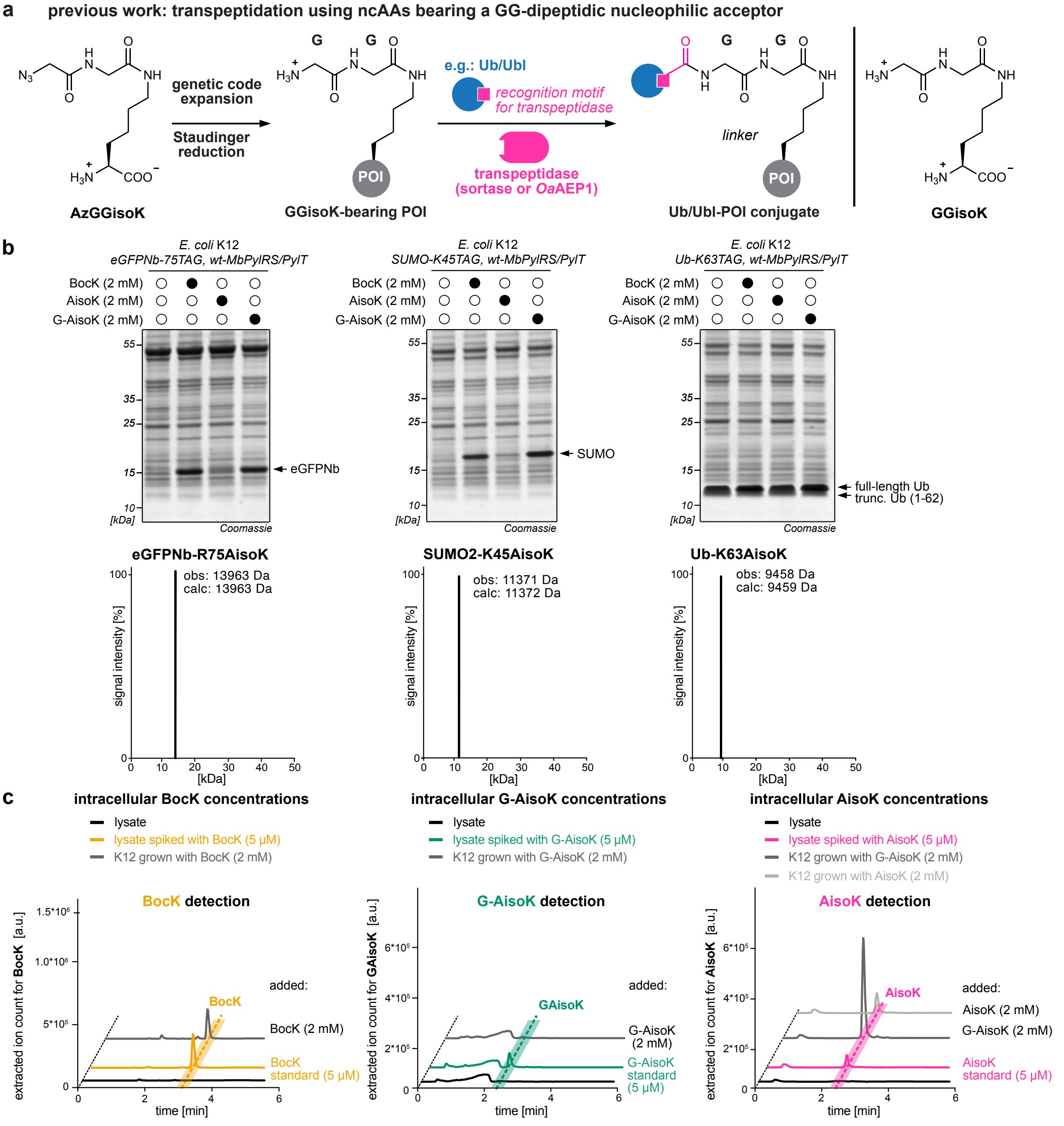

Supplementary Figure 1: Previous work and genetic encoding of AisoK in the presence of G-AisoK into different POIs. **a.** Previously reported ncAA AzGGisoK (left) is site-specifically incorporated into proteins via GCE and decaged on protein via Staudinger reduction to yield GGisoK-modified POIs. The dipeptidic GG acceptor nucleophile can subsequently engage in transpeptidation with proteins bearing a recognition motif at their C-termini using sortase or *Oa*AEP1 to create protein-protein conjugates. Right: structure of GGisoK. **b**. Top: SDS-PAGE analysis of eGFP nanobody (eGFPNb-R75TAG), SUMO2-K45TAG and Ubiquitin (Ub-K63TAG) expression using wt-*Mb*PylRS/PylT in the presence of BocK, AisoK and G-AisoK. Very efficient full-length protein expression was observed when cells were supplemented with G-AisoK (comparable to BocK incorporation). Bottom: LC-MS analysis of all three proteins purified from cells supplemented with G-AisoK revealed specific AisoK incorporation. **c**. Extracted ion chromatograms of *E. coli* lysates to measure intracellular concentrations of BocK (left), G-AisoK (middle) and AisoK (right). Cells grown in the presence of 2 mM BocK showed low intracellular BocK concentrations. Cells grown in presence of 2 mM G-AisoK showed no detectable intracellular G-AisoK (middle). In contrast, intracellular AisoK concentrations were up to 10-fold higher (right, dark grey) when cells were supplemented with G-AisoK as compared to cells grown in 2 mM AisoK (right, light grey).

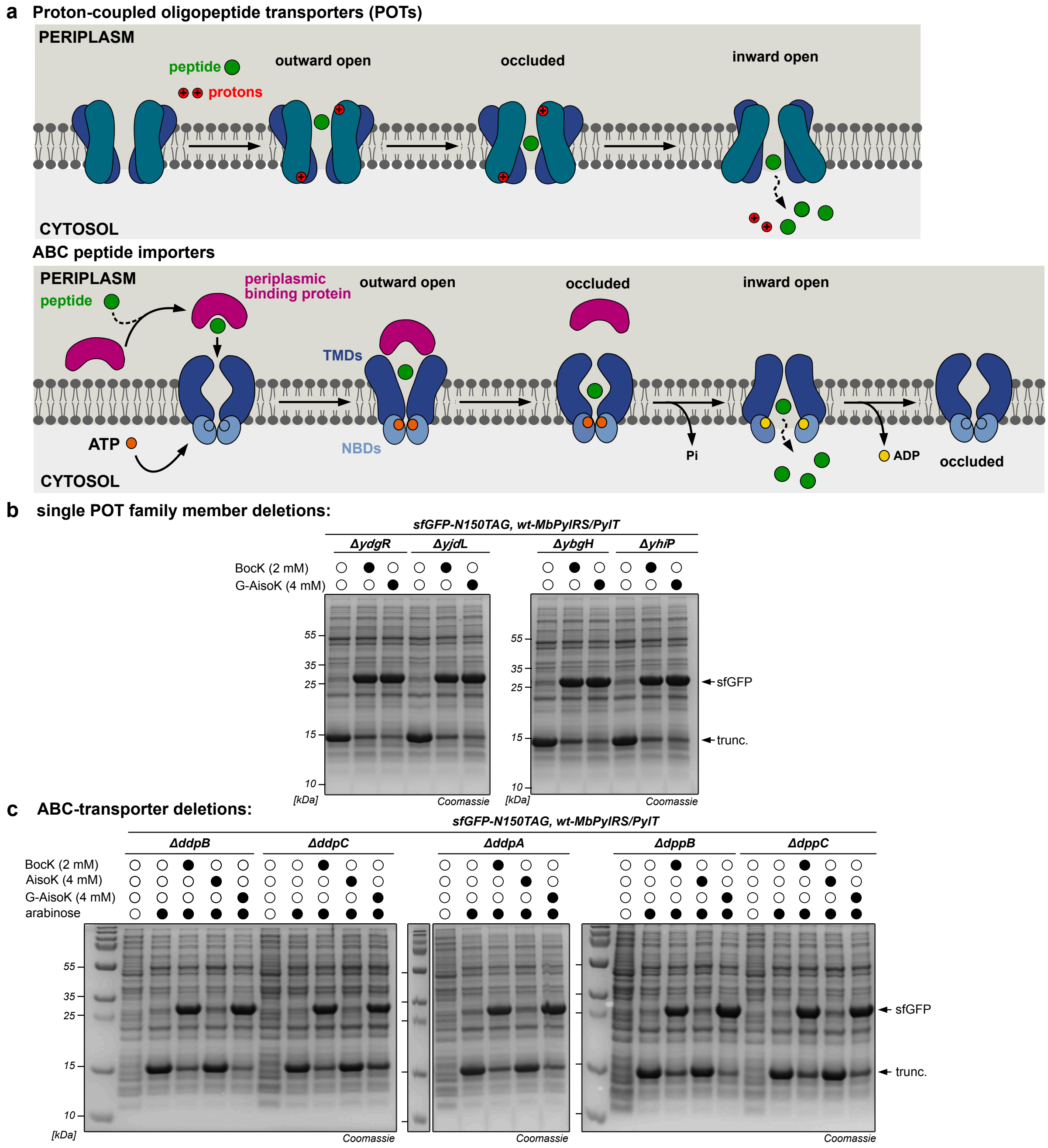

Supplementary Figure 2: Identifying bacterial transporter systems for G-AisoK uptake. **a.** Top: scheme illustrating the transport mechanism of Proton-coupled oligopeptide transporters (POTs). Peptides are co-transported along a proton gradient into the cell. Bottom: scheme illustrating the mechanism of ATP-binding cassette (ABC) peptide importers. A periplasmic binding protein binds to and shuttles the peptide to the periplasm-facing site of the transmembrane domains (TMDs) (outward open conformation), which triggers ATP binding and dimerization of the two cytoplasmic nucleotide-binding domains (NBDs) that bind and hydrolyze ATP, leading to uptake of the peptide into the translocation channel (occluded conformation). Hydrolysis of ATP leads to conformational changes in the NBDs and dissociation of their dimerization interface and flips the TMDs to trigger release of the substrate peptide into the cytoplasm (inward open state). Once the peptidic ligand is shuffled into the cytosol, the apo form of the periplasmic binding protein is released and can bind to another peptide. **b.** Single gene knockouts (KOs) of POT family members *ydg*R, *yjd*L, *ybg*H and *yhi*P. None of these KOs abolished the expression of full-length sfGFP-N150TAG in the presence of G-AisoK. **c.** Single gene KOs of other oligopeptide ABC transporters *ddp*A,B,C (D,D-dipeptide permease specific towards D-ala-D-ala peptides) and *dpp*B,C (dipeptide permease specific towards dipeptides). None of these KOs abolished expression of full-length sfGFP-N150TAG in the presence of G-AisoK.

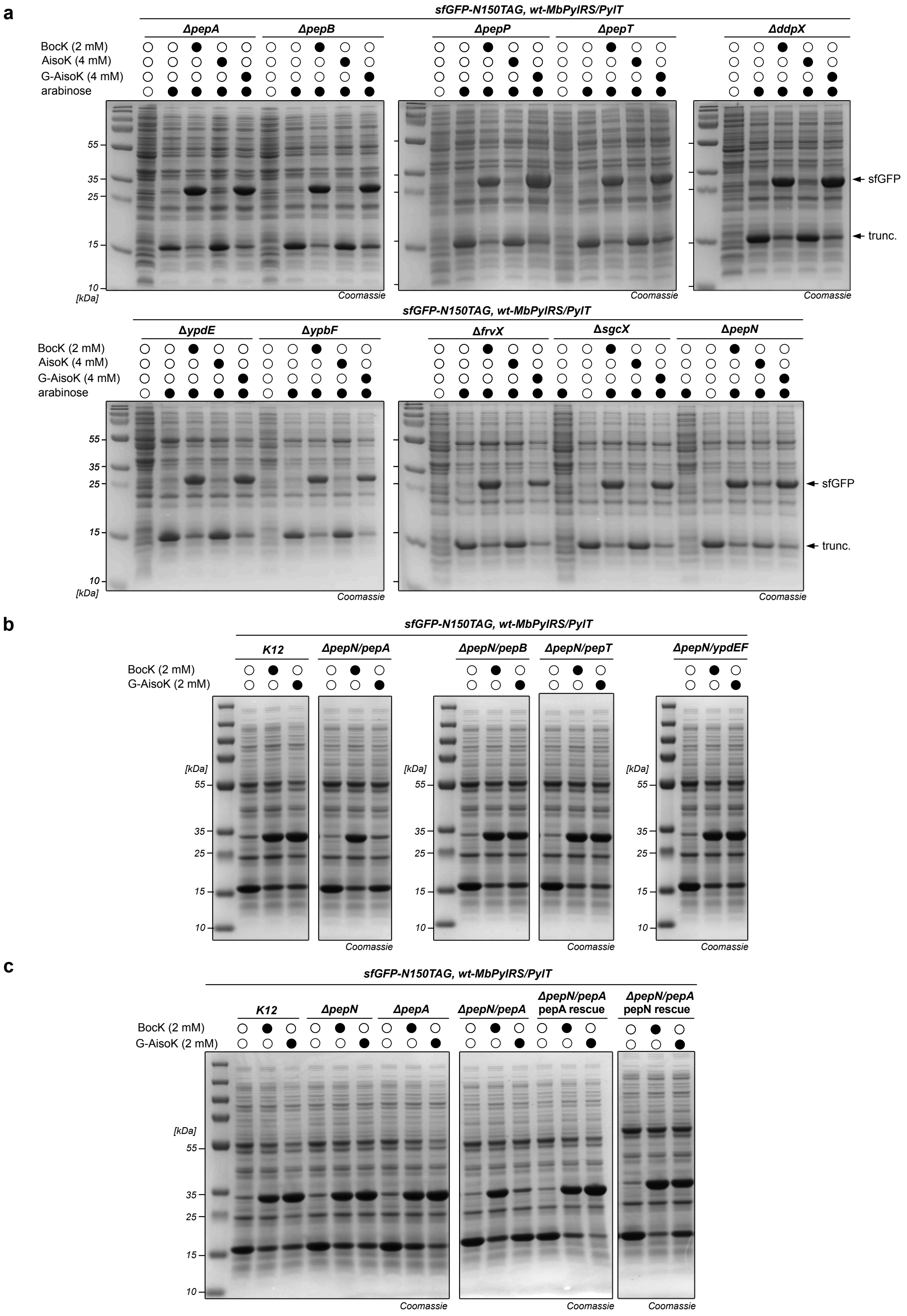

Supplementary Figure 3: Identifying the peptidase(s) responsible for N-terminal glycine cleavage. **a.** SDS-PAGE analysis of sfGFP-N150TAG expression in K12 knockout cell lines that had individually single aminopeptidase enzymes *pep*A, *pep*B, *pep*P, *pep*T, *ddp*X, *ypd*E, *ypb*F, *frv*X, *sgc*X and *pep*N genomically deleted. None of these KOs abolished expression of full-length sfGFP-N150TAG in the presence of G-AisoK and wt-*Mb*PylRS/PylT, suggesting there is no single peptidase that is solely responsible for the cleavage of the N-terminal glycine of G-AisoK. **b.** SDS-PAGE analysis of sfGFP-N150TAG expression in K12 multi-knockout

cell lines with peptidases *pepN* along with either *pepA*, *pepB, pepT* or *ypdE* and *ypdF* knocked out. Full-length sfGFP expression is significantly diminished in the double knockout *ΔpepN/pepA*. Other double knockouts had no significant effect on full-length sfGFP expression. **c.** SDS-PAGE analysis of sfGFP-N150TAG expression in wt-K12 compared to *ΔpepA, ΔpepN* and *ΔpepN/pepA*. A rescue system with pepA or pepN expressed via a plasmid in *ΔpepN/pepA* cells restores full-length sfGFP expression levels.

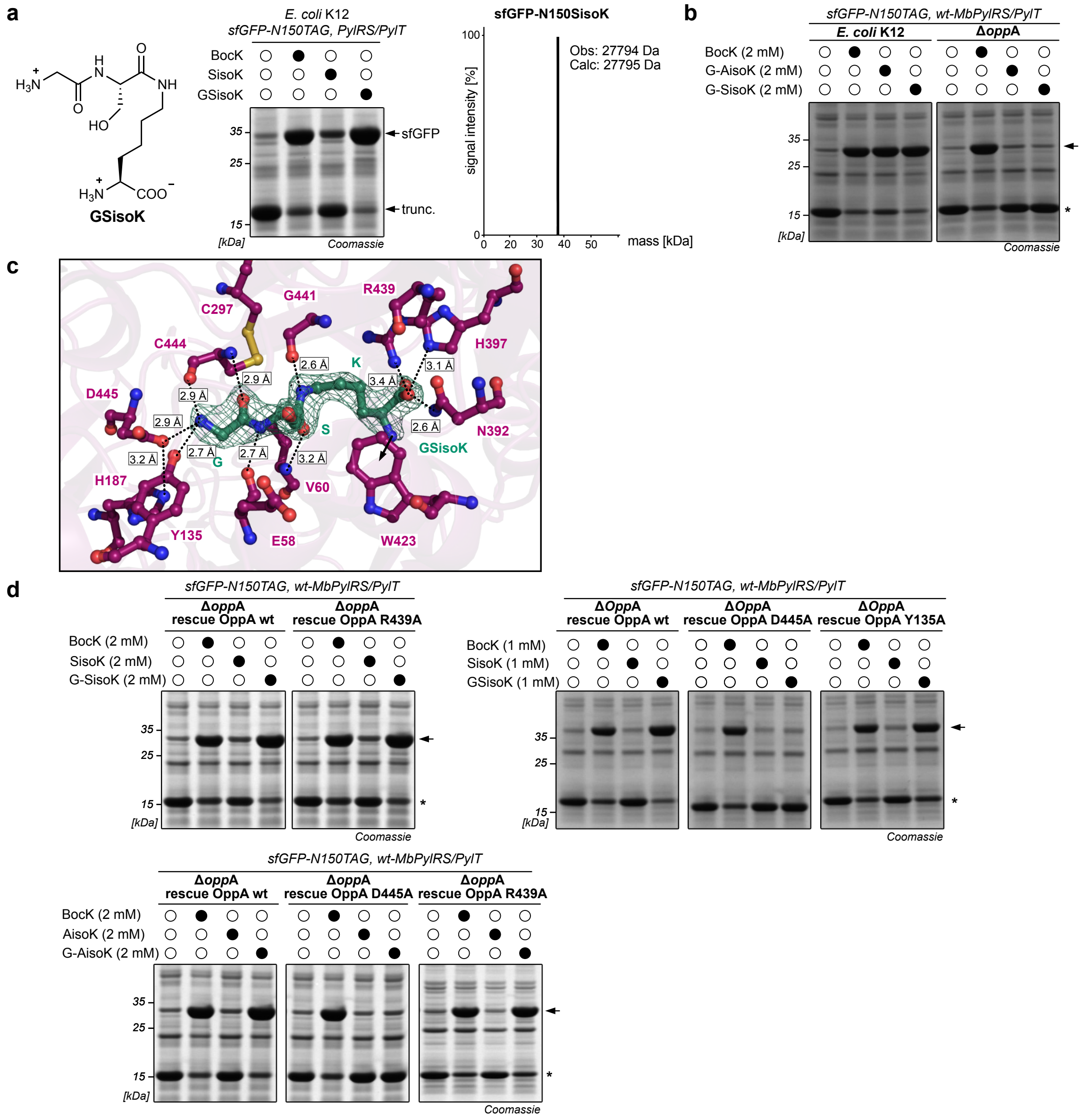

Supplementary Figure 4: a. Structure of G-SisoK (left). SDS-PAGE analysis of sfGFP-N150TAG expression in cells grown in the presence of BocK, SisoK and G-SisoK (middle). Similarly to G-AisoK, expression in the presence of G-SisoK leads to high levels of full-length sfGFP expression, while its dipeptide counterpart, SisoK, shows a low level of expression. LC-MS analysis of purified sfGFP-N150TAG expressed in the presence of G-SisoK reveals sfGFP bearing SisoK. **b.** SDS-PAGE analysis of sfGFP-N150TAG expression in K12 compared to Δ*oppA* cells. Knocking out *opp*A completely abolishes full-length sfGFP expression in the presence of G-AisoK and G-SisoK. **c.** X-ray crystal structure of wt-OppA:G-SisoK (PDB ID: 9RD1). OppA residues interact with G-SisoK by targeting the ligand's backbone and termini. The N-terminal glycine in G-SisoK makes multiple interactions with OppA residues and is tightly kept in place by an extensive network of hydrogen bonds and salt bridges. Specifically, the protonated α-amine of glycine is in hydrogen bonding distance to the side chains of D445 and Y135, as well as the backbone carbonyl of C444. The carboxyl side chain of D445 is in hydrogen-bonding distance to H187, keeping it in its deprotonated form and facilitating salt bridge formation with the positively charged amine of G in G-SisoK. The backbone carbonyl of G in G-SisoK hydrogen-bonds to the backbone amine of C444 in OppA. The C-terminus of G-SisoK is hold in place by hydrogen bonding involving the side chains of R439, H397, N392 as well as cation-π interactions with W423. Further hydrogen interactions of the backbone amides of G-SisoK can be seen with back bone moieties of E58, V60 and G441. **d.** SDS-PAGE analysis of sfGFP-N150TAG expression in Δ*oppA* cells with wt-OppA and single point mutants R439A, D445A and Y136A expressed via a plasmid. Mutations R439A and Y135A have no effect on full-length sfGFP expression in the presence of G-AisoK or G-SisoK. Mutation D445A completely abolishes full-length sfGFP expression. Arrows indicate full-length sfGFP, asterisks indicate truncated sfGFP.

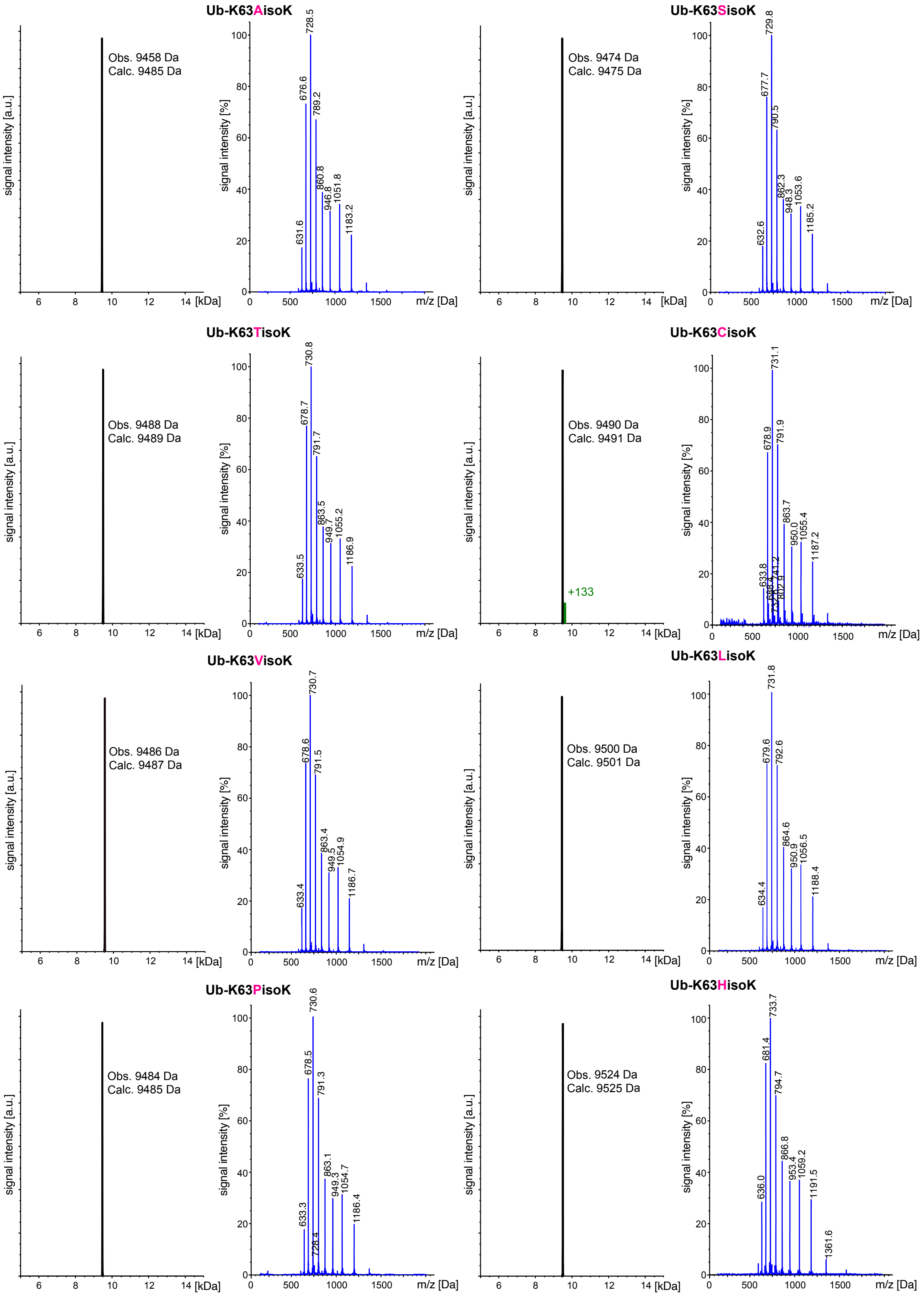

Supplementary Figure 5. LC-MS analysis of purified Ub-K63XisoK expressed in the presence of G-XisoK derivatives, in which X stands for a natural amino acid. All observed masses confirm incorporation of the corresponding XisoK derivatives. Ub-K63CisoK was treated with methoxyamine to remove metabolic adducts.

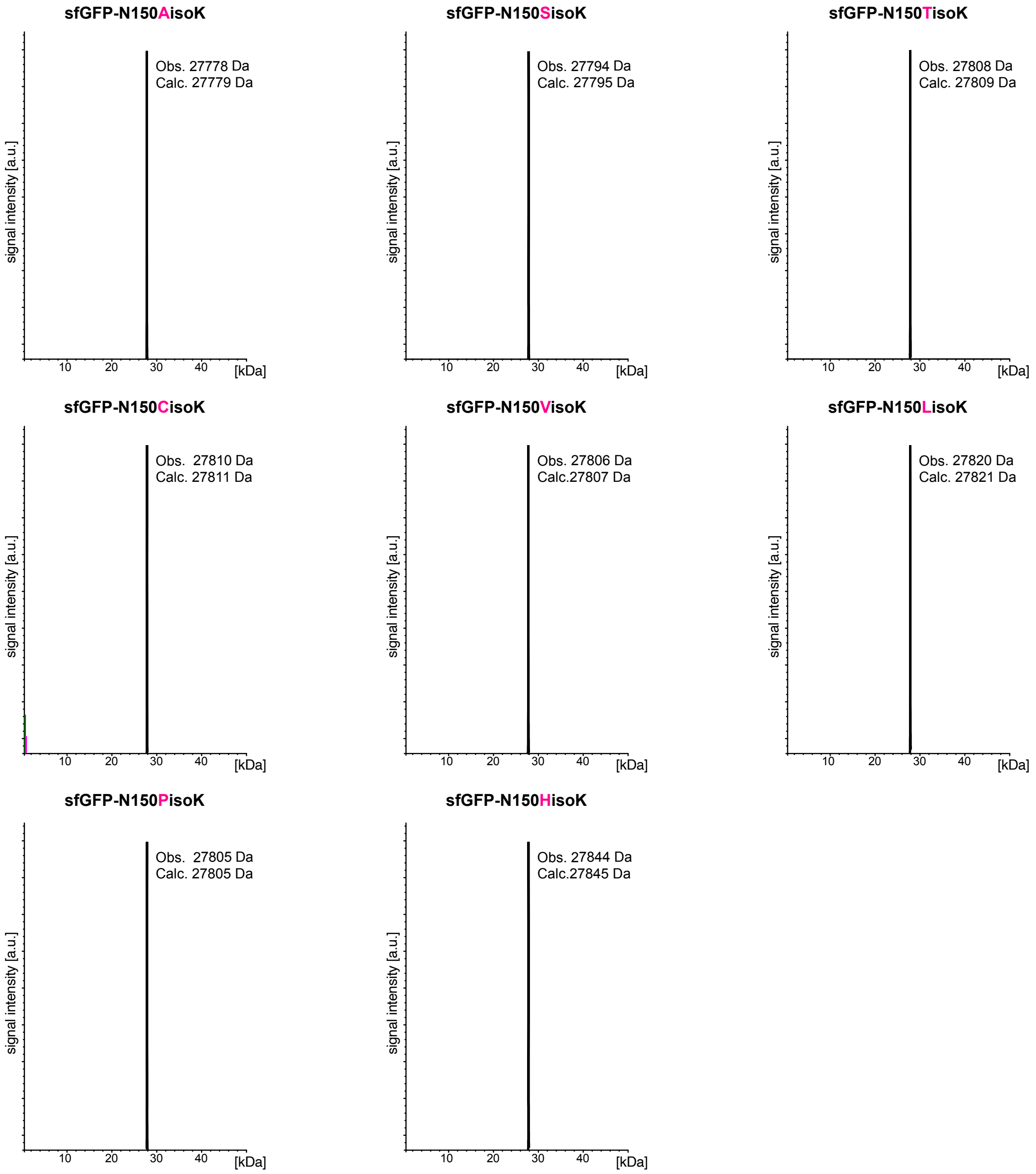

Supplementary Figure 6: LC-MS analysis of purified sfGFP-N150XisoK expressed in the presence of G-XisoK derivatives, where X is a natural amino acid. All observed masses confirm incorporation of the corresponding XisoK derivatives into sfGFP. sfGFP-N150CisoK was treated with methoxyamine to remove metabolic adducts.

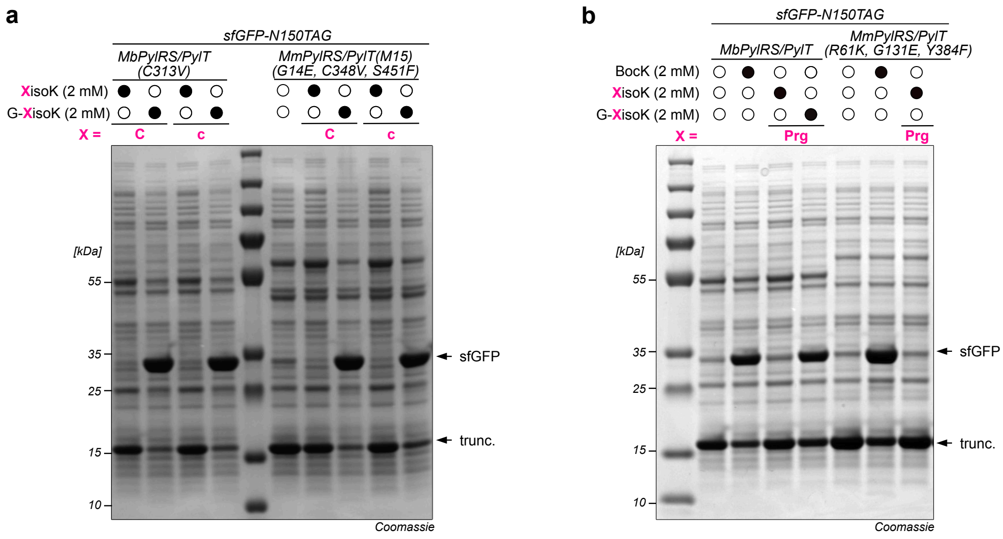

Supplementary Figure 7: Comparison of incorporation efficiencies using the G-XisoK scaffold versus previously reported evolved PylRS variants for CisoK and PrgisoK (C = L-cysteine, c = D-cysteine). **a**. SDS-PAGE analysis of sfGFP-N150TAG expression with G-CisoK, CisoK, G-cisoK and cisoK using *Mb*PylRS(C313V)/PylT used in this study or a previously reported aaRS/tRNA pair for cisoK incorporation (*Mm*PylRS(G14E/C348V/S451F)/ PylT(M15[1])[2]. Similar expression yields for full-length sfGFP are obtained for G-CisoK and G-cisoK with both aaRS/tRNA pairs, while nearly no full-length sfGFP expression is observed using the dipeptides CisoK/cisoK and either of the aaRS/tRNA pairs, indicating the superiority our tripeptide-based approach. **b.** SDS-PAGE analysis of sfGFP-N150TAG expression with BocK, PrgisoK and G-PrgisoK comparing wt-*Mb*PylRS/PylT used in this study with a previously reported PylRS/tRNA pair for PrgisoK incorporation (*Mb*PylRS(R61K/G131E/Y348F) /PylT)[3]. Full-length sfGFP expression is significantly higher using G-PrgisoK and wt-*Mb*PylRS/PylT in comparison to the previously published synthetases with dipeptide PrgisoK.

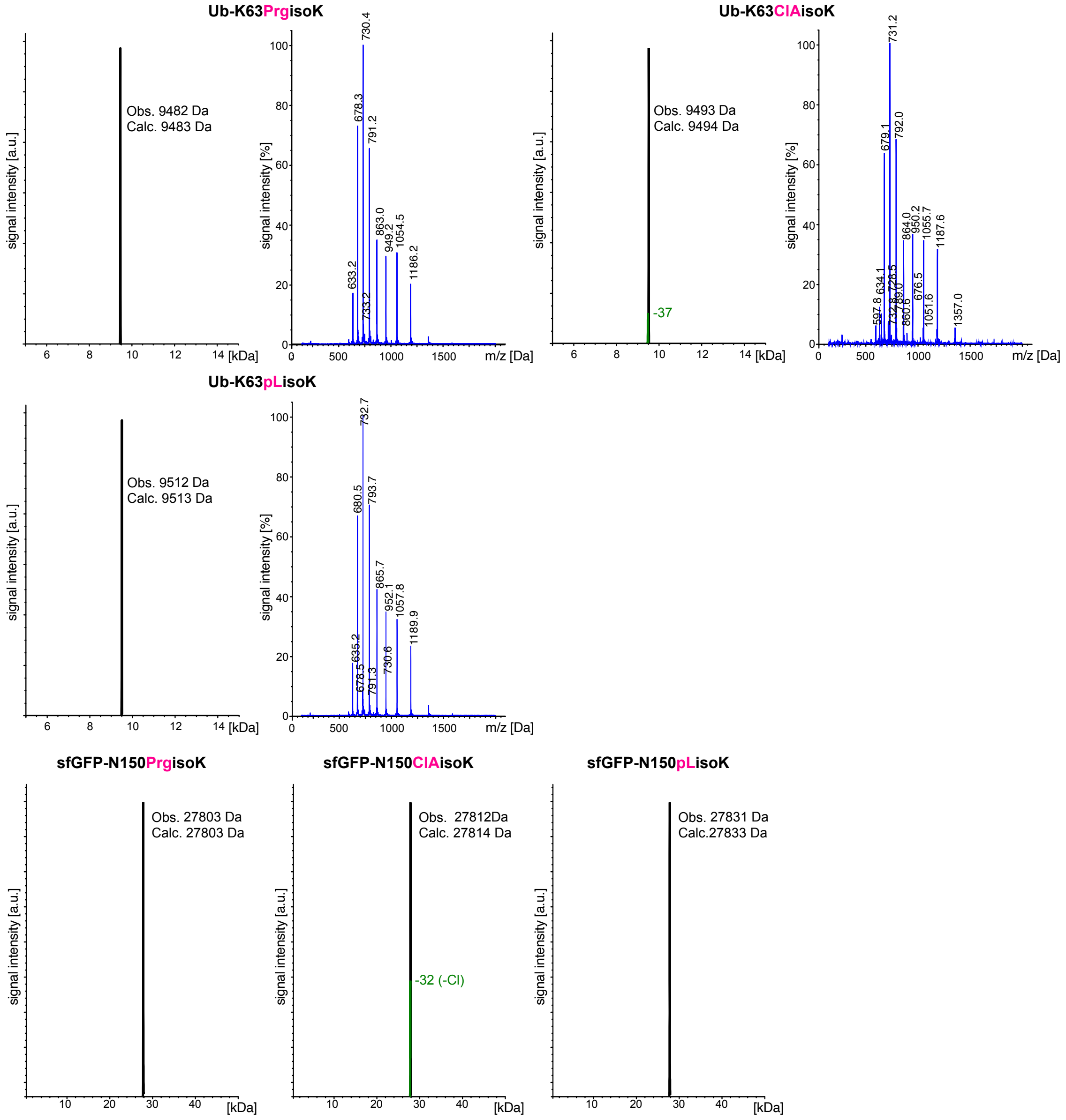

Supplementary Figure 8: LC-MS analysis of purified Ub-K63XisoK and sfGFP-N150XisoK expressed in the presence of G-XisoK derivatives, where X is an ncAA. Observed masses confirm incorporation of the corresponding XisoK derivatives. Peaks denoted in green for Ub-K63ClAisoK and sfGFP-N150ClAisoK correspond to the elimination of HCl.

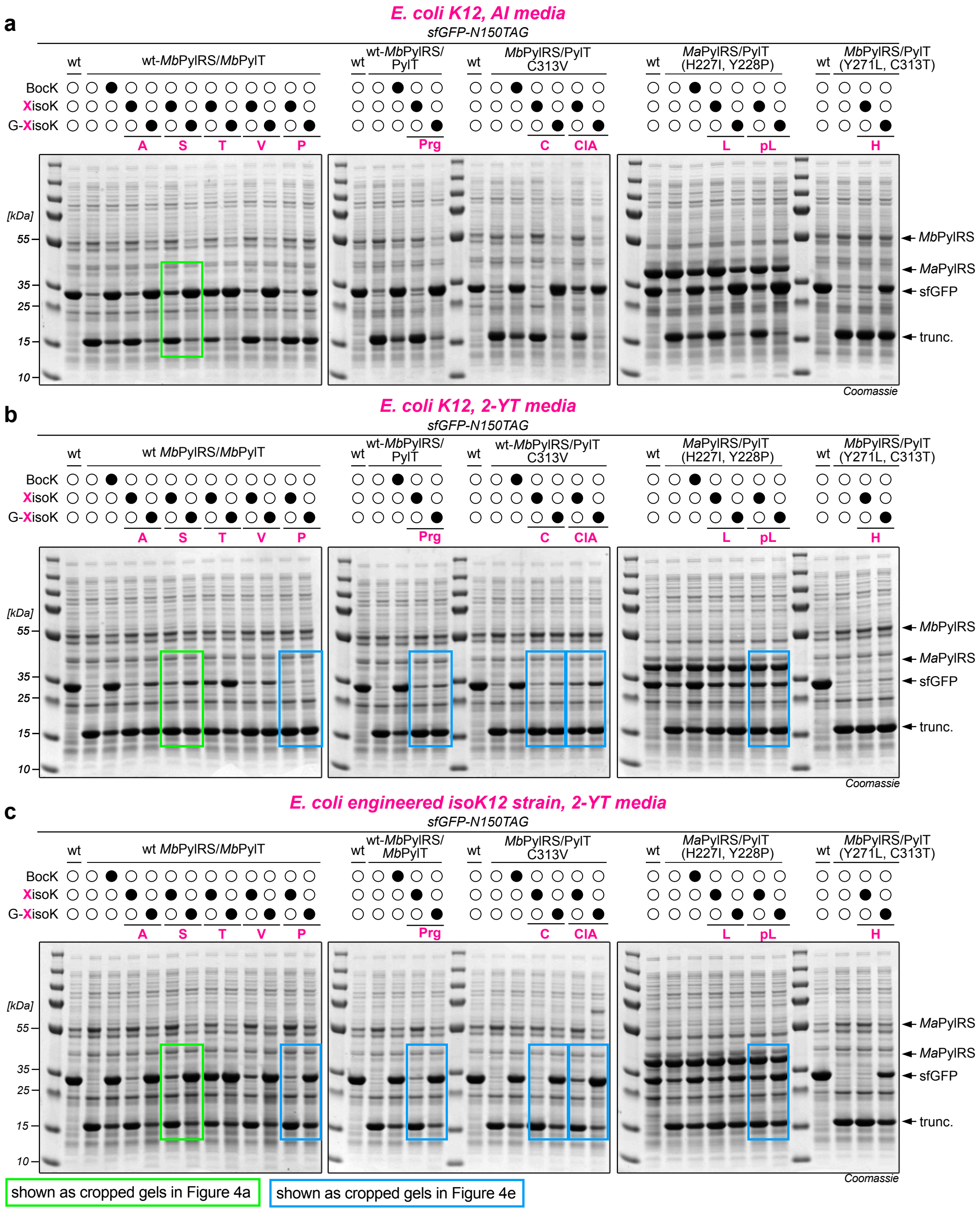

Supplementary Figure 9: SDS-PAGE analysis of sfGFP-N150TAG expression in the presence 2 mM of all G-XisoK and XisoK derivatives used in this study, comparing amber suppression levels in K12 cells versus engineered IsoK12 cells grown in different media. **a.** Expression in K12 cells using autoinducing (AI) media. **b.** Expression in K12 cells using 2-YT media. **c.** Expression in engineered IsoK12 cells using 2-YT media.

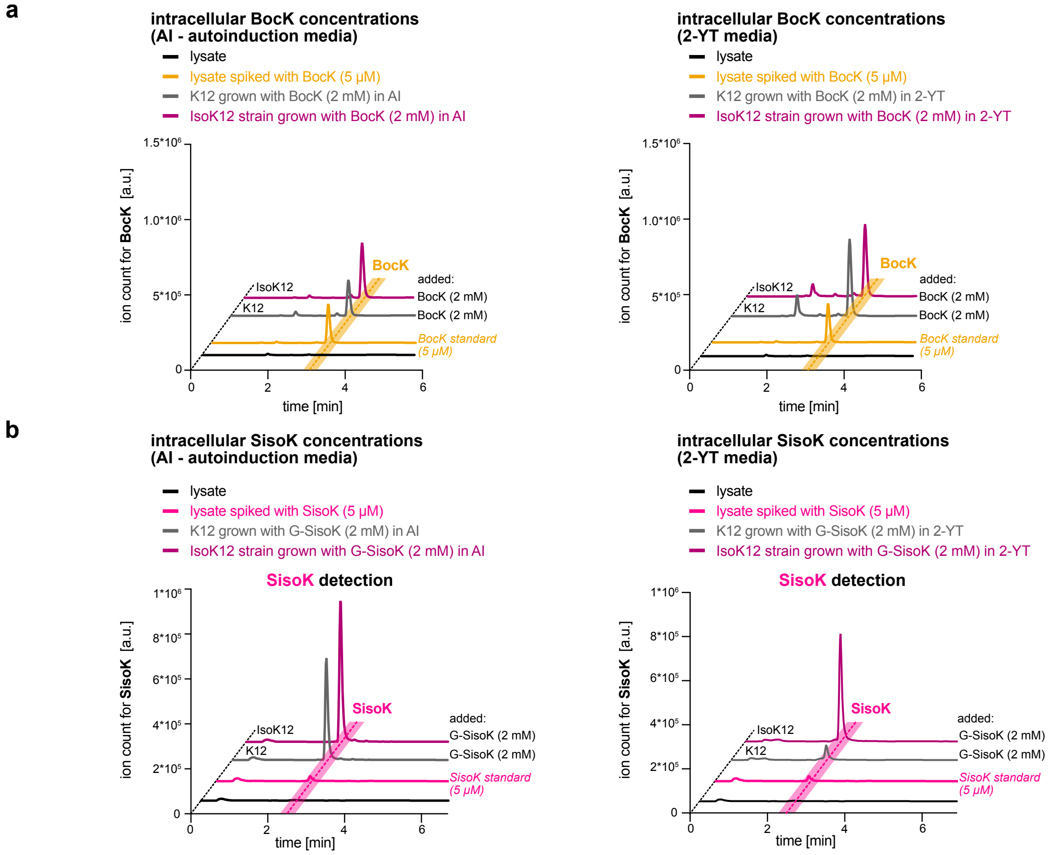

Supplementary Figure 10: Extracted ion chromatograms of *E. coli* lysates to determine intracellular concentrations of ncAAs. **a.** Extracted ion chromatograms to determine intracellular BocK concentrations. wt-K12 cells or IsoK12 cells are grown in presence of 2 mM BocK using AI media (left) or 2-YT media (right). BocK concentrations in lysate are comparable for both cell types in both media. **b.** Extracted ion chromatograms to determine intracellular SisoK concentrations. wt-K12 cells or IsoK12 cells grown in presence of 2 mM G-SisoK using AI media (left) or 2-YT media (right). SisoK concentrations in AI media are ca. 1.4-fold higher in IsoK12 cells compared to wt-K12 cells (left). In 2-YT media, SisoK concentrations are 7-10-fold higher in IsoK12 cells (purple) compared to wt-K12 cells (grey) (right).

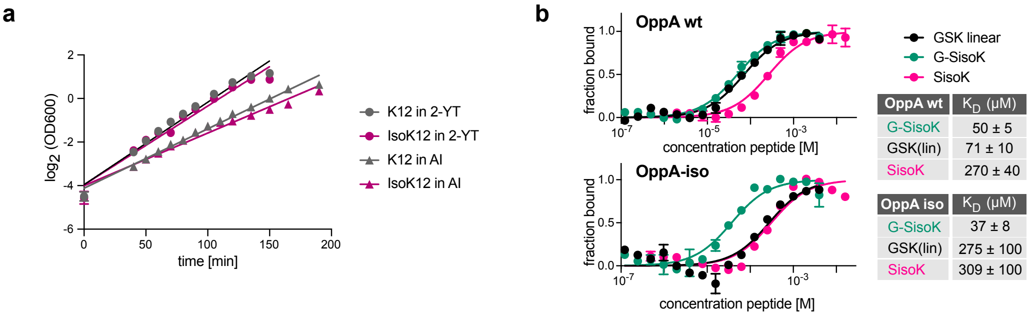

Supplementary Figure 11: Characterization of evolved IsoK12 strain. **a.** Doubling times of wt-K12 cells (grey) and IsoK12 cells (purple) grown in AI media and 2-YT. Doubling times for IsoK12 in 2-YT was 19 minutes compared to K12, which was 18 minutes. In AI media, IsoK12 had a doubling time of 29 minutes in comparison to K12, which was 25 minutes. **b.** Microscale thermophoresis affinity measurements for GSK linear (GSK(lin)), G-SisoK and SisoK towards wt-OppA and evolved OppA-iso. Affinity towards SisoK and G-SisoK remained similar for both wt-OppA and OppA-iso (50 µM vs 37 µM for G-SisoK and 270 µM vs 309 µM for SisoK). In contrast affinity towards a linear GSK tripeptide is reduced ~ 4-fold (71 µM vs 275 µM) for OppA-iso.

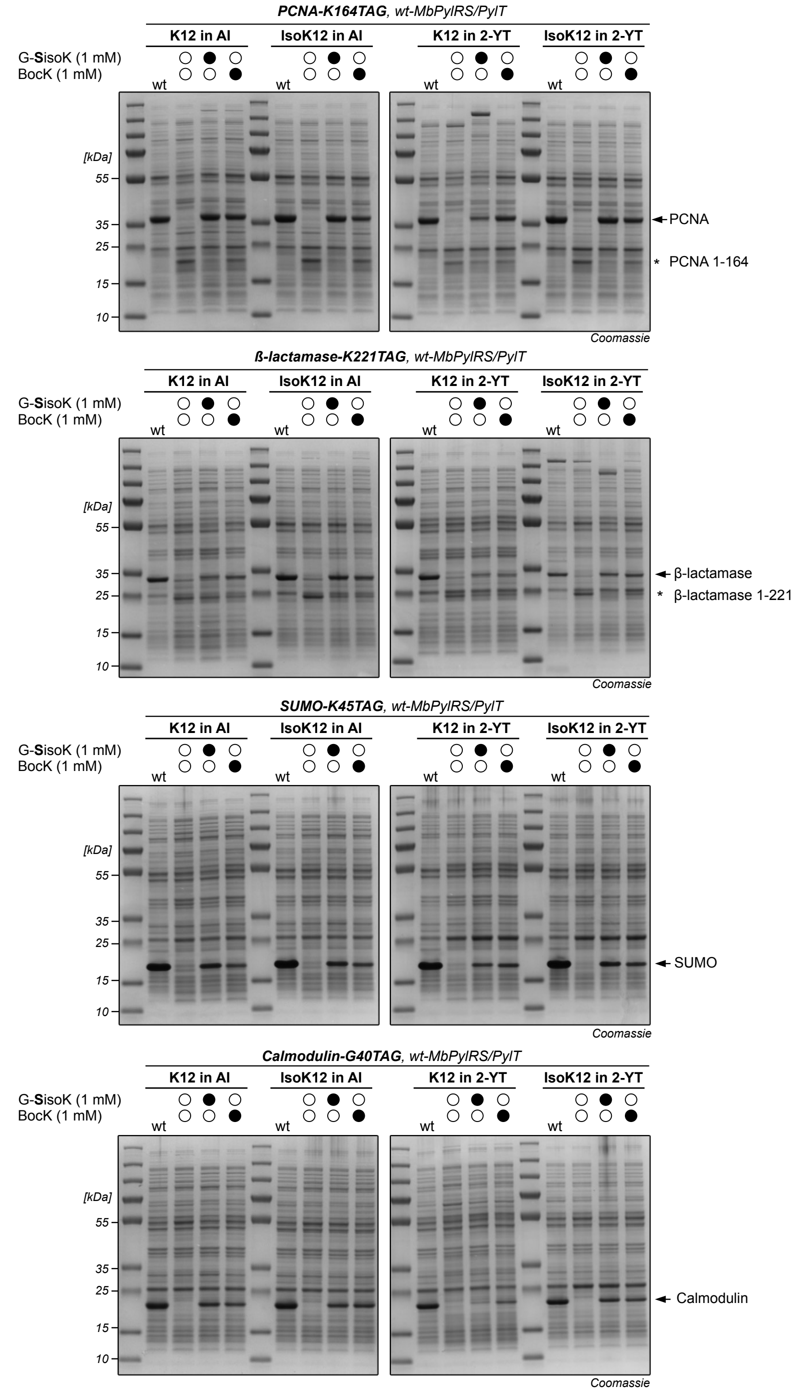

Supplementary Figure 12: SDS-PAGE analysis of amber suppression efficiencies within different POIs in the presence of 1 mM BocK or G-SisoK in wt-K12 and IsoK12 cells comparing AI media with 2-YT. From the top: PCNA-K164TAG, β-lactamase-K122TAG, SUMO-K45TAG and Calmodulin-G40TAG. In all cases, amber suppression efficiency for G-SisoK is considerably higher in IsoK12 cells than in wt-K12 cells grown in 2-YT. Arrows indicate full-length POIs, asterisks indicate truncated proteins.

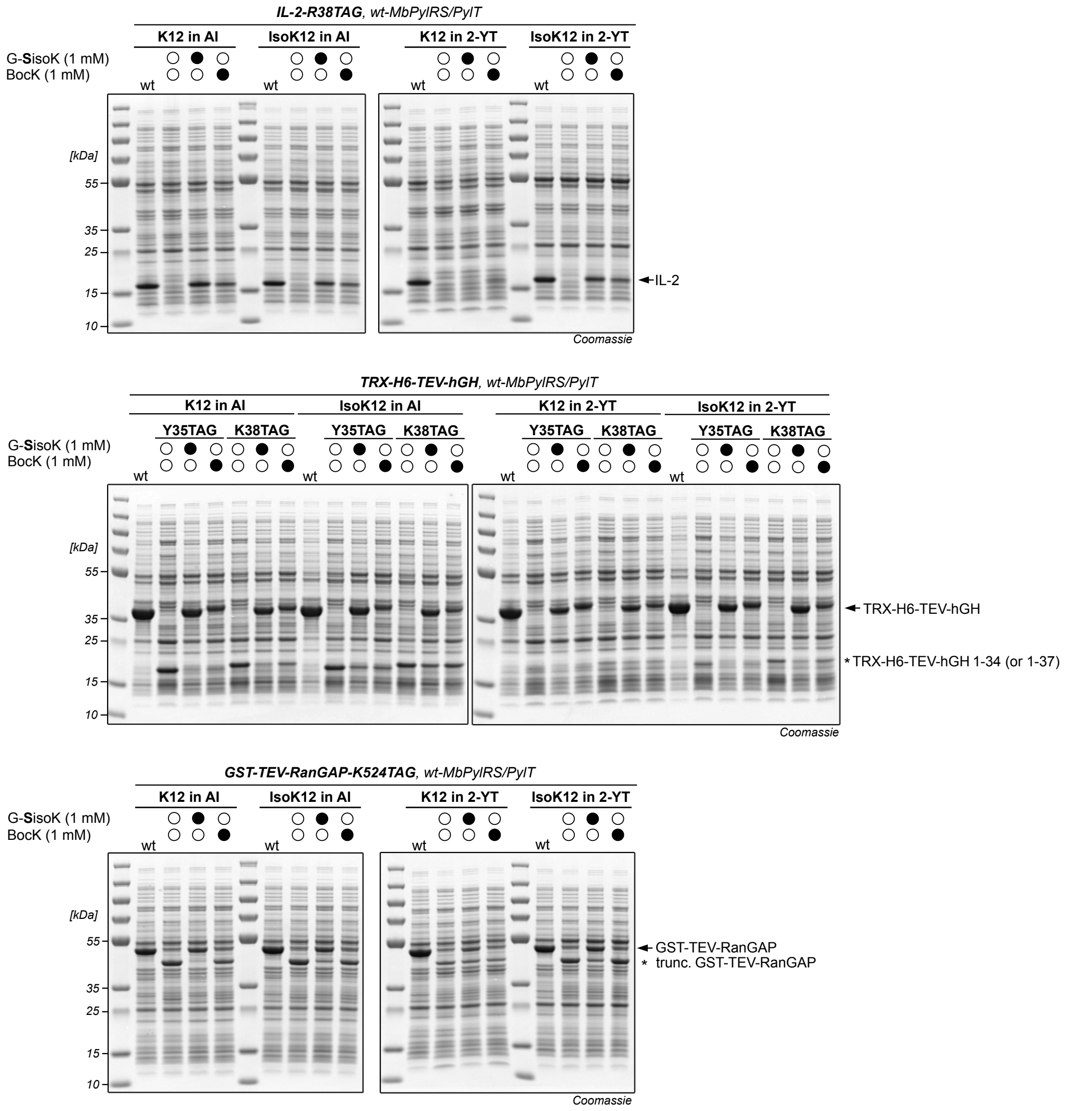

Supplementary Figure 13: SDS-PAGE analysis of amber suppression efficiencies within different POIs in the presence of 1 mM BocK or G-SisoK in wt-K12 and IsoK12 cells comparing AI media with 2-YT. From the top: Interleukin-2 (IL-2)-R38TAG, Human Growth Factor (hGH)-Y35TG and K38TAG and RanGAP K524TAG. In all cases, amber suppression efficiency for G-SisoK is considerably higher in IsoK12 cells than in wt-K12 cells grown in 2-YT. Arrows indicate full-length POIs, asterisks indicate truncated proteins.

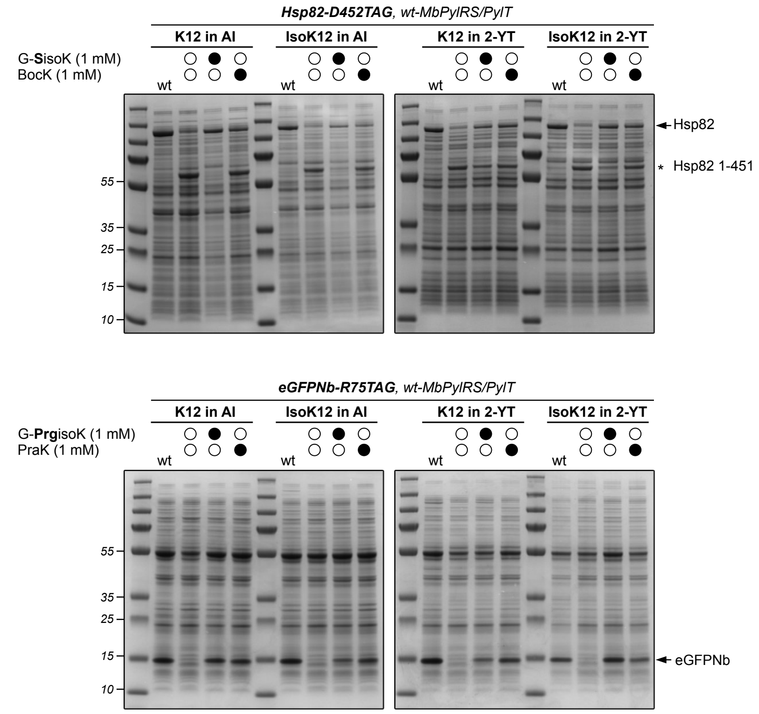

Supplementary Figure 14: a. SDS-PAGE analysis of amber suppression efficiencies within different POIs in the presence of 1 mM BocK, G-SisoK, PraK[4] or G-PrgisoK in wt-K12 and IsoK12 cells comparing AI media and 2-YT media. Top: Hsp82-D452TAG, Bottom: eGFPNb-R75TAG. Full-length protein expression is higher in IsoK12 cells compared to wt-K12 cells in 2-YT.

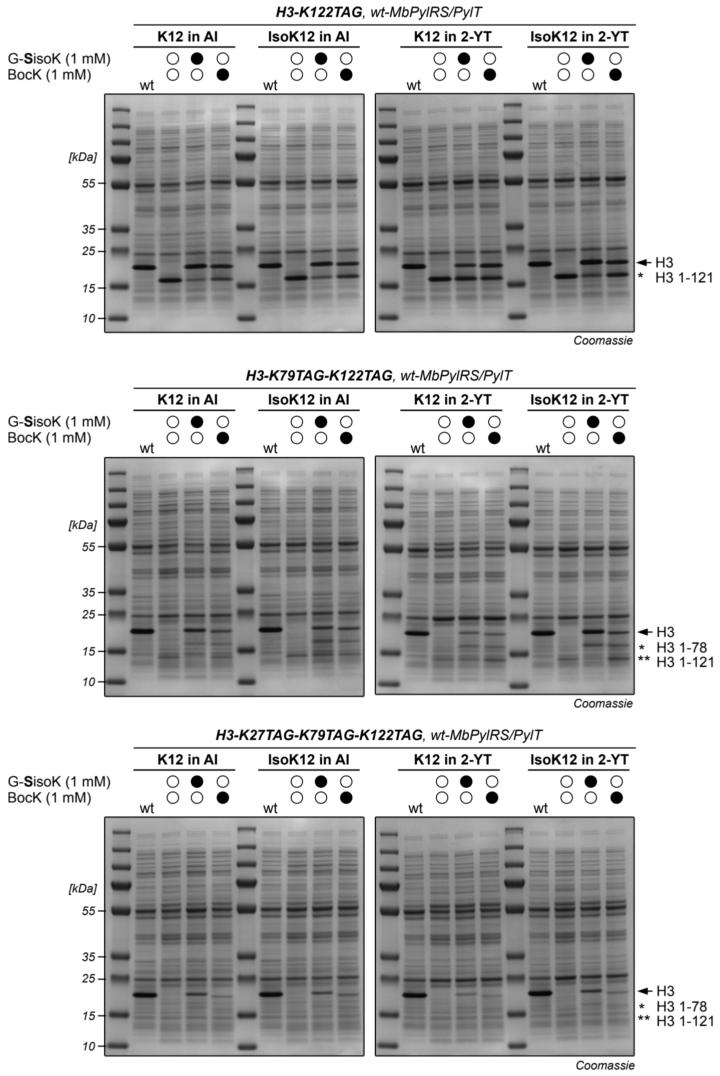

Supplementary Figure 15: SDS-PAGE analysis of amber suppression efficiencies for Histone H3 variants bearing either 1 TAG codon (K122TAG, top), 2 TAG codons (K79TAG, K122TAG, middle) or 3 TAG codons (K27TAG, K79TAG, K122TAG, bottom) in wt-K12 or IsoK12 cells, comparing expression in AI media or 2-YT media. Expression of all variants are higher in IsoK12 in 2-YT. Arrows indicate full-length POIs, asterisks indicate truncated proteins.

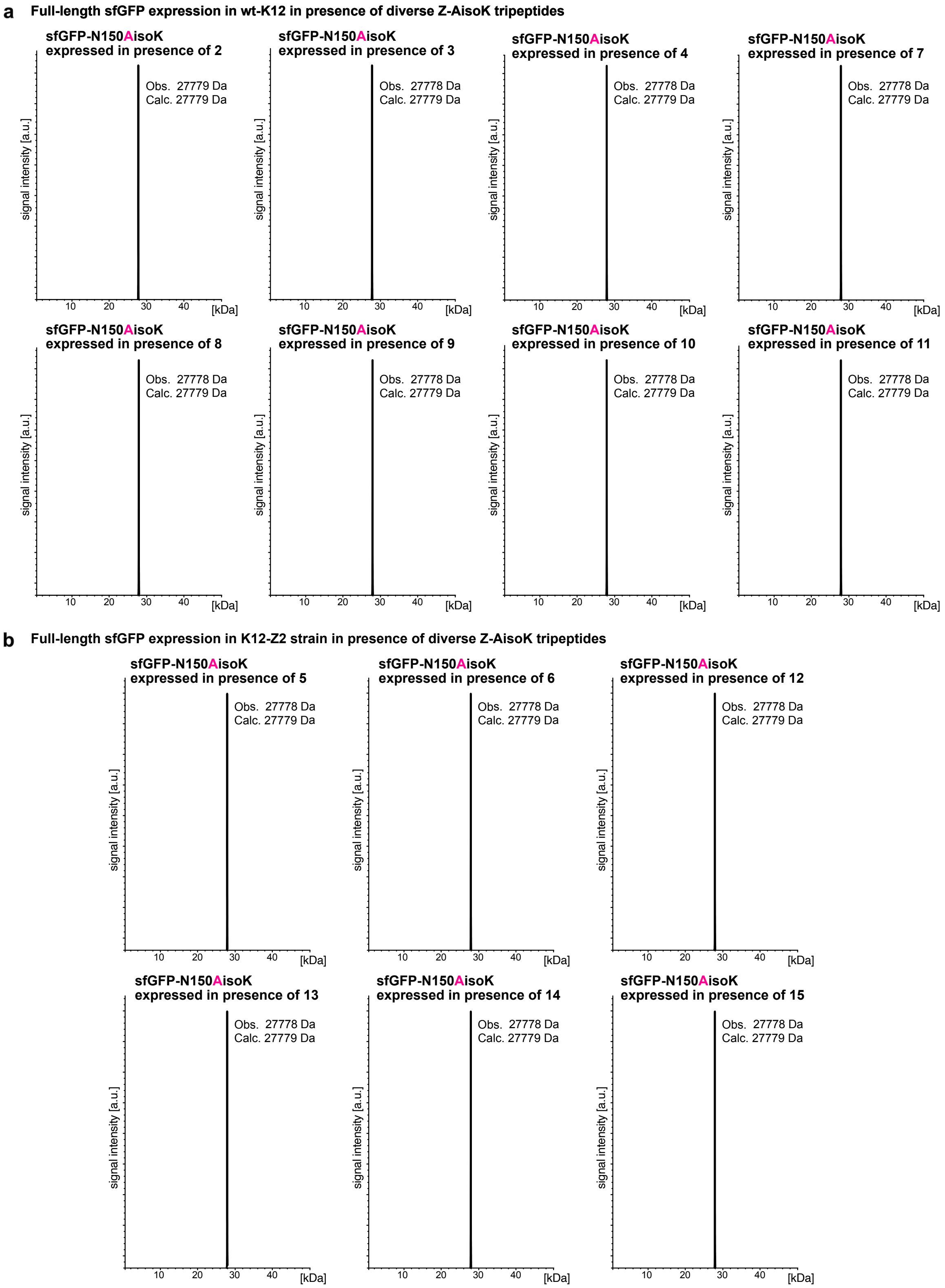

Supplementary Figure 16: LC-MS analysis of purified sfGFP expressed in the presence of Z-AisoK peptides with wt-*Mb*PylRS/PylT. **a.** sfGFP expressed in wt-K12 cells in the presence of Z-AisoK peptides 1, 2, 3, 4, 6, 7, 8, 9, 10 and 11. **b.** sfGFP expressed in K12-Z2 in the presence of Z-AisoK peptides 5, 6, 12, 13, 14 and 15. All observed masses confirm the incorporation of AisoK.

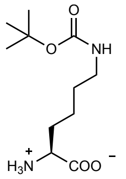
BocK

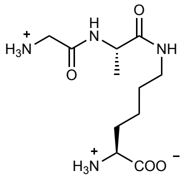
G-AisoK (1)

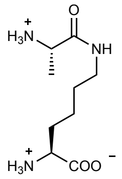
AisoK

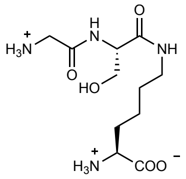
G-SisoK

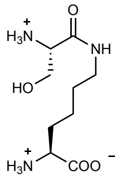
SisoK

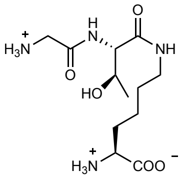
G-TisoK

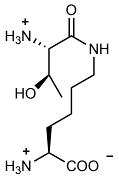
TisoK

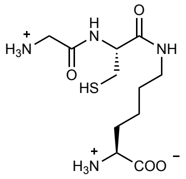
G-CisoK

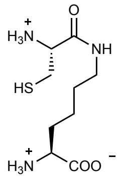
CisoK

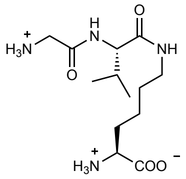
G-VisoK

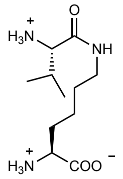
VisoK

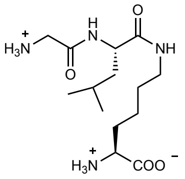
G-LisoK

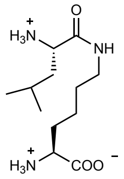
LisoK

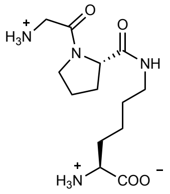
G-PisoK

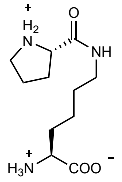
PisoK

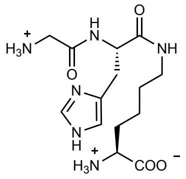
G-HisoK

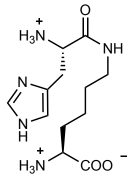
HisoK

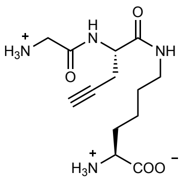
G-PrgisoK

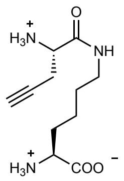
PrgisoK

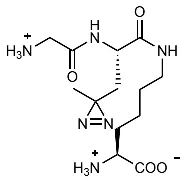
G-pLisoK

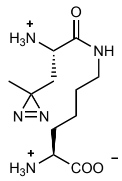
pLisoK

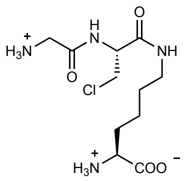
G-ClAisoK

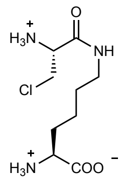
ClAisoK

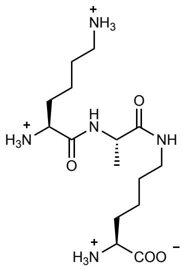
K-AisoK (2)

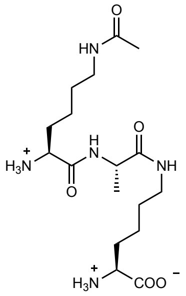
AcK-AisoK (3)

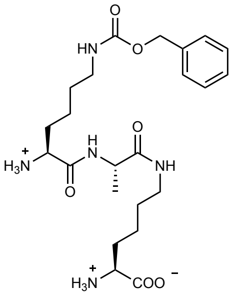
CbzK-AisoK (4)

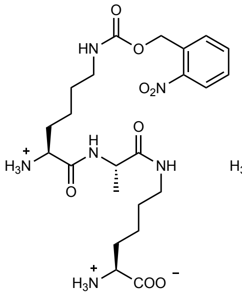
ONBK-AisoK (5)

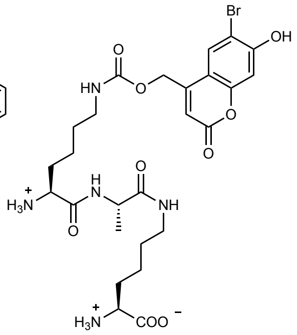
CouK-AisoK (6)

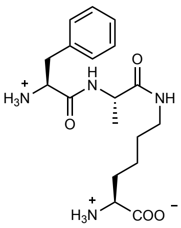
F-AisoK (7)

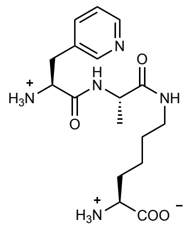
3PyA-AisoK (8)

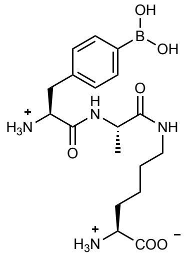
BoF-AisoK (9)

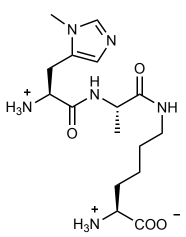
3mH-AisoK (10)

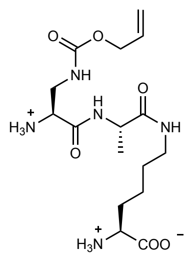
AllocDAP-AisoK (11)

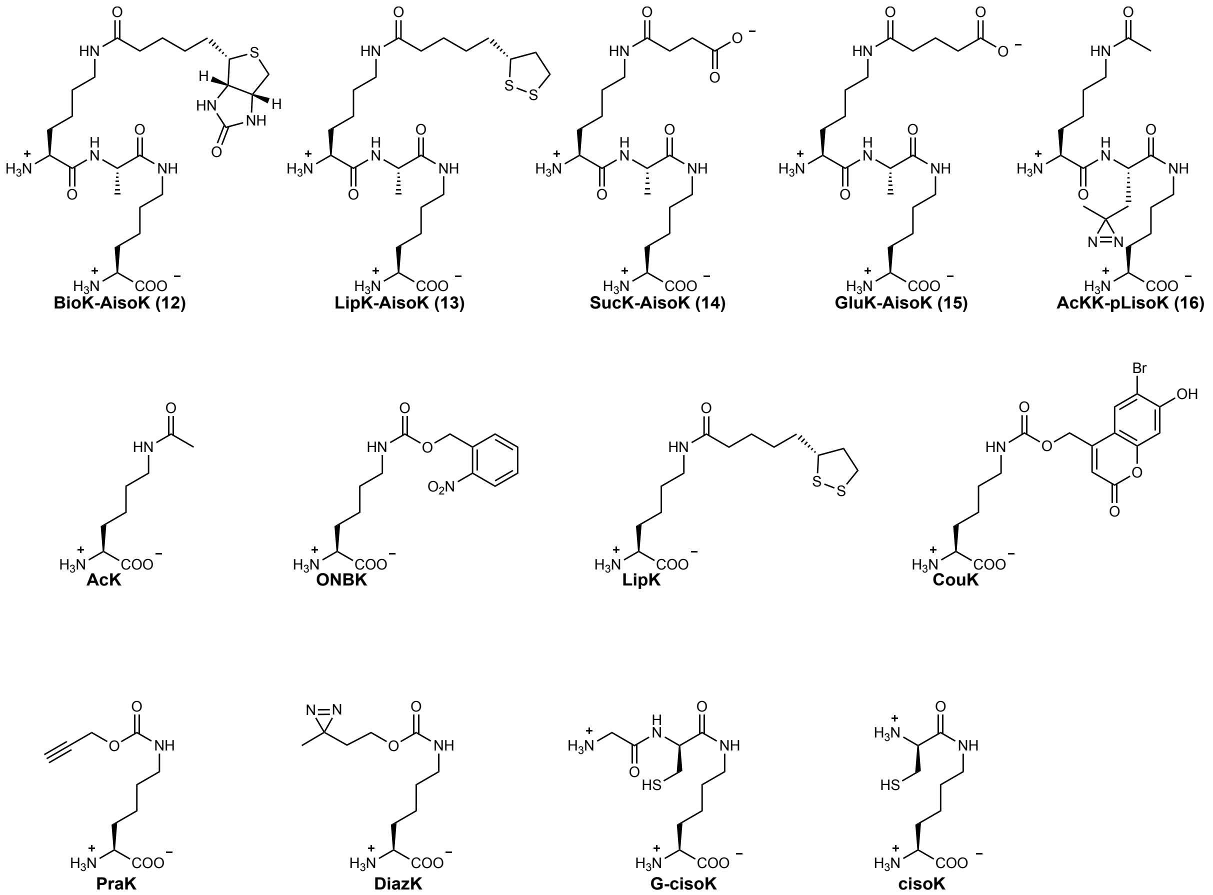

Supplementary Figure 17: Structures of all peptides/ncAAs used in this study.

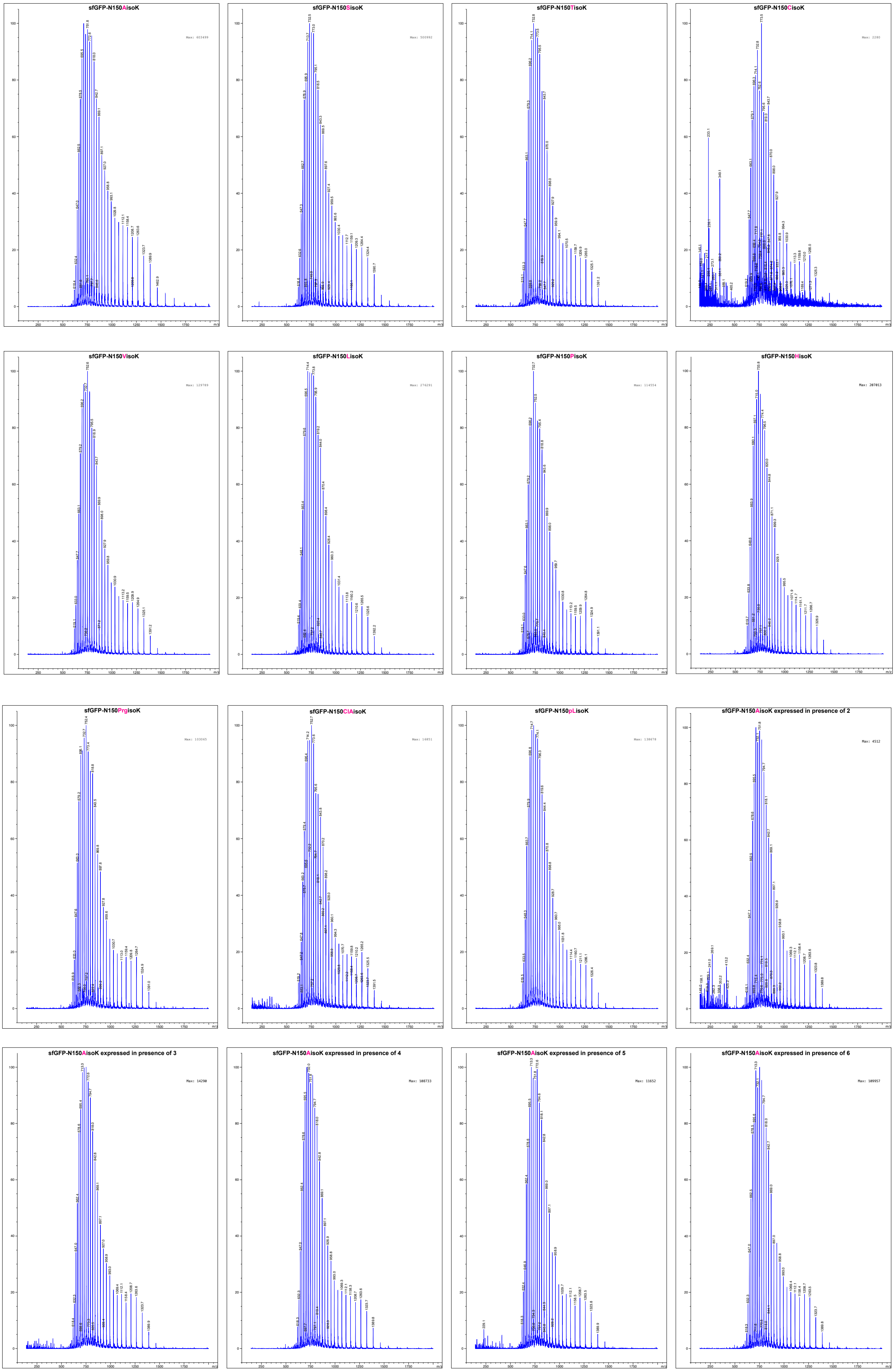
sfGFP-N150AisoK
sfGFP-N150SisoK
sfGFP-N150TisoK
sfGFP-N150CisoK
sfGFP-N150VisoK
sfGFP-N150LisoK
sfGFP-N150PisoK
sfGFP-N150HisoK
sfGFP-N150PrgisoK
sfGFP-N150ClAisoK
sfGFP-N150pLisoK
sfGFP-N150AisoK expressed in presence of 2
sfGFP-N150AisoK expressed in presence of 3
sfGFP-N150AisoK expressed in presence of 4
sfGFP-N150AisoK expressed in presence of 5
sfGFP-N150AisoK expressed in presence of 6

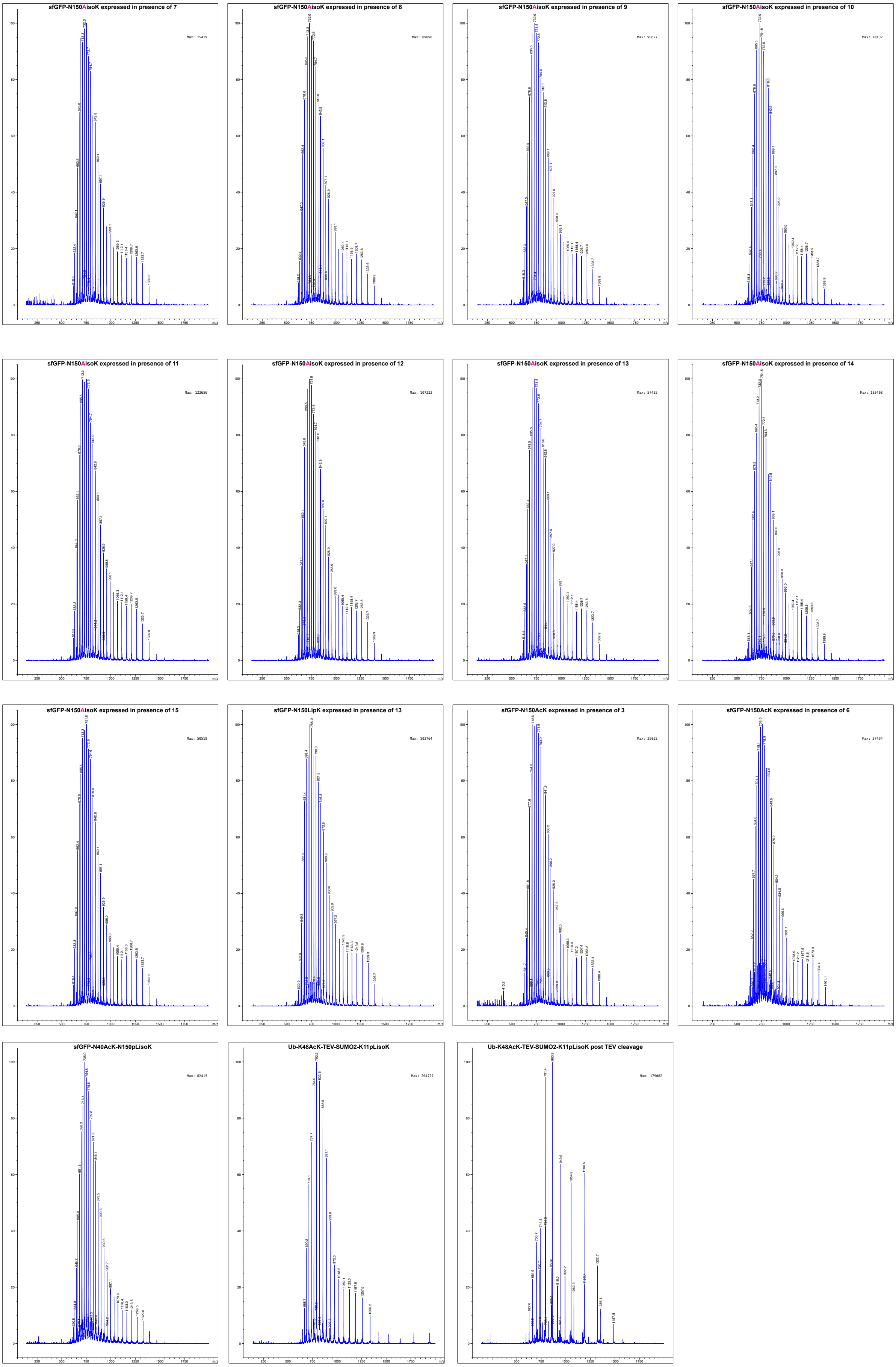

Supplementary Figure 18: m/z spectra of proteins in this study.

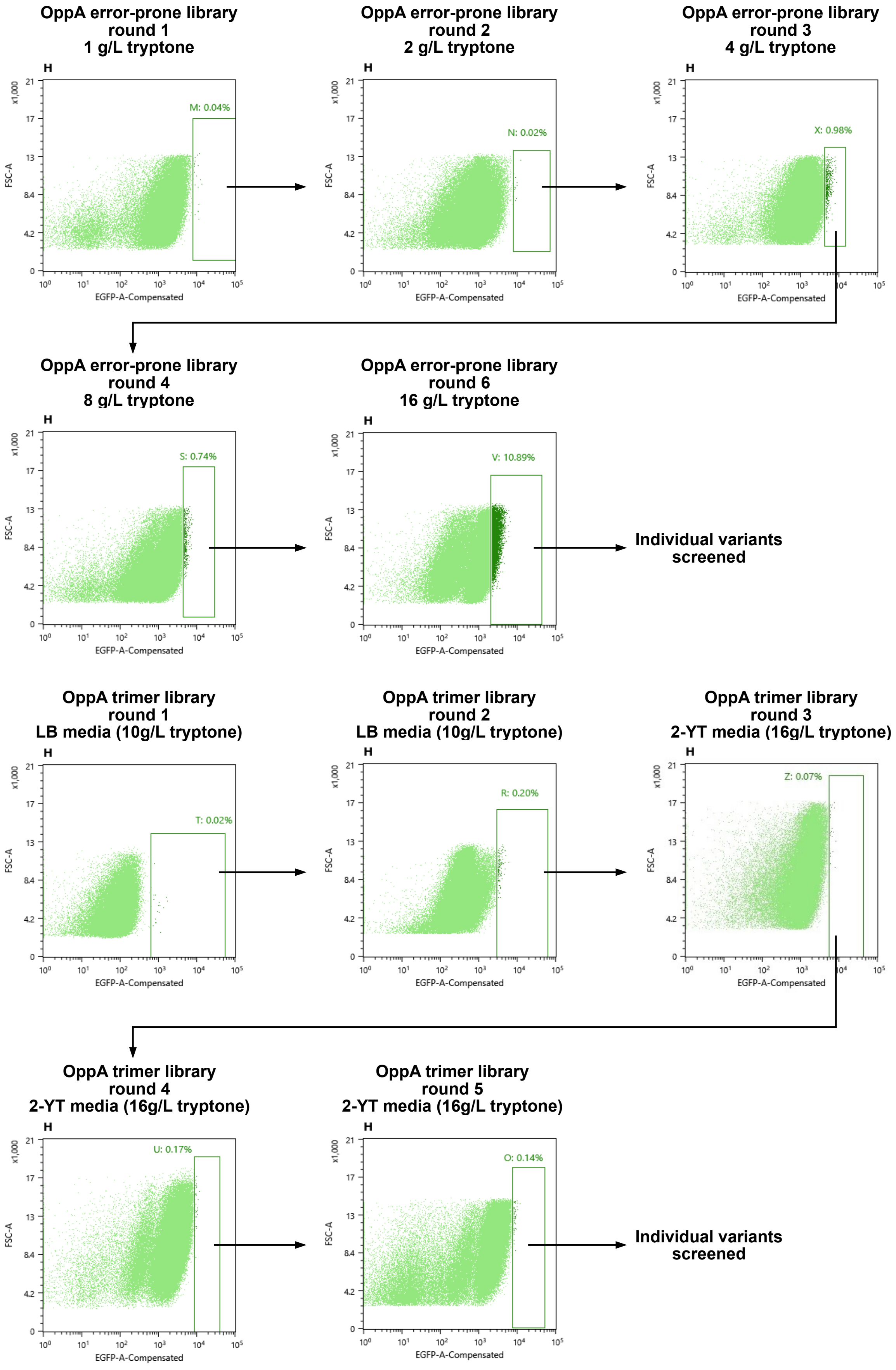

Supplementary Figure 19: FACS data plots and gating strategies for the sorting of error prone and site-saturation trimer library for the evolution of OppA-iso.

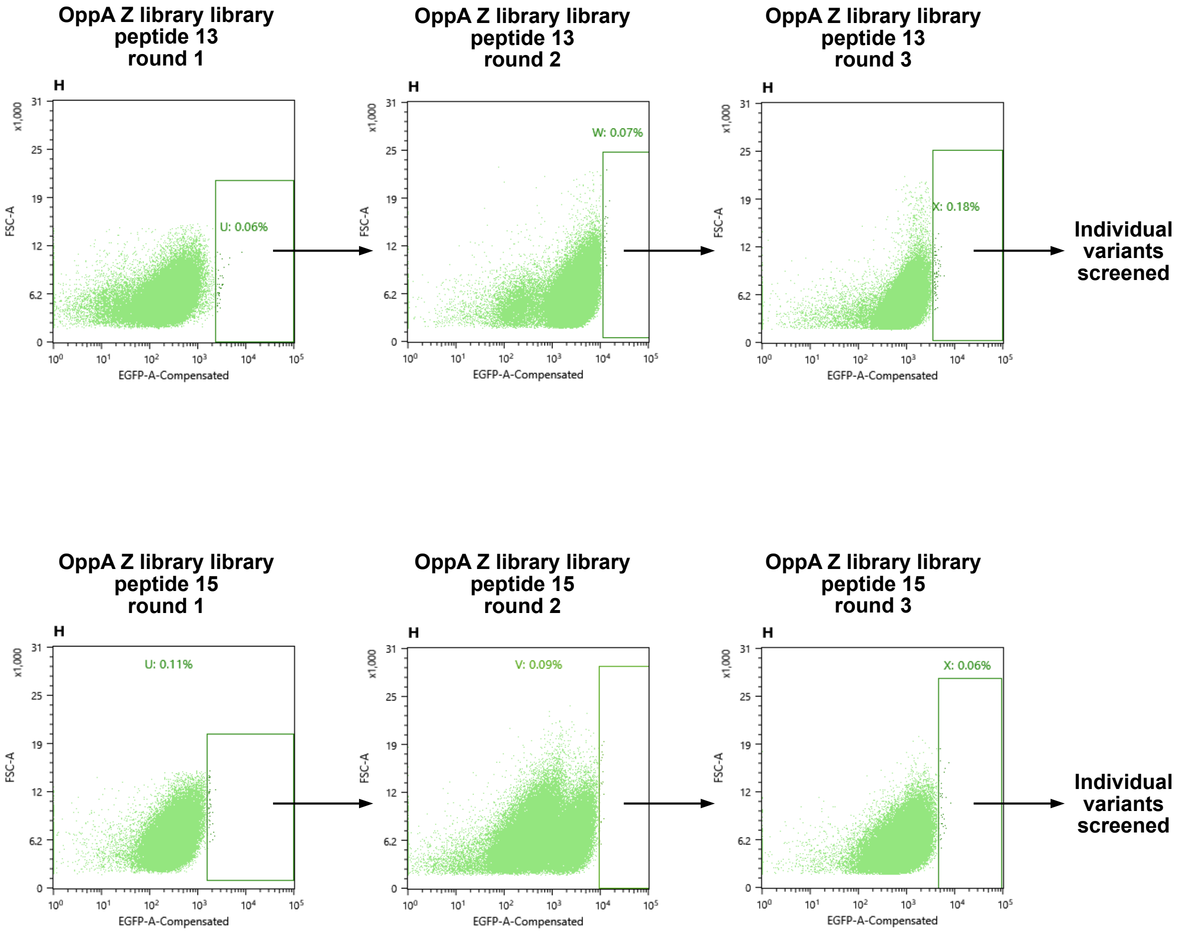

Supplementary Figure 20: FACS data plots and gating strategies for the sorting of site saturation Z-library for the evolution of OppA-Z1 (top) and OppA-Z2 (bottom).

red box shown as a cropped gel in Fig. 1b

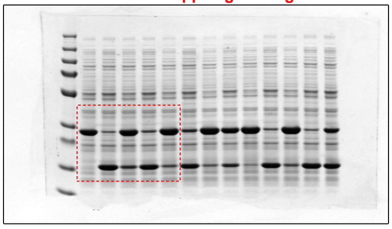

red boxes are shown as a cropped gels in Fig. 2a and Extended. Fig. 1a

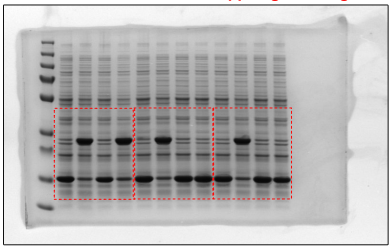

red boxes are shown as a cropped gels in Fig. 2a and Extended. Fig. 1a

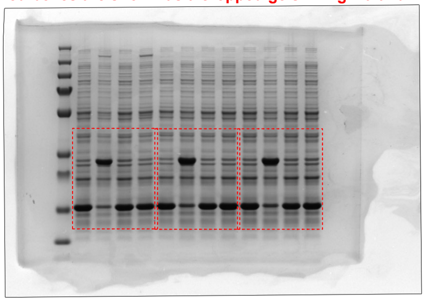

red boxes are shown as a cropped gels in Fig. 2d and S3c

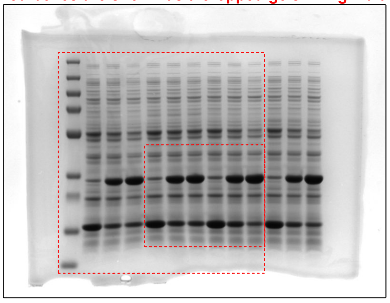

red boxes are shown as a cropped gels in Fig. 2d and S3c

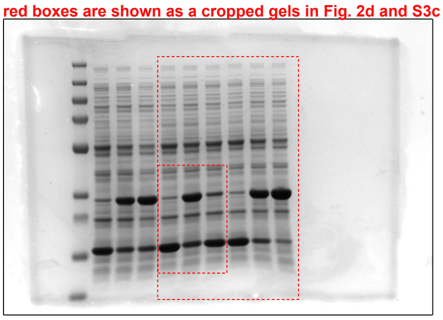

red boxes are shown as a cropped gels in Fig. 3e, 4a and S9a

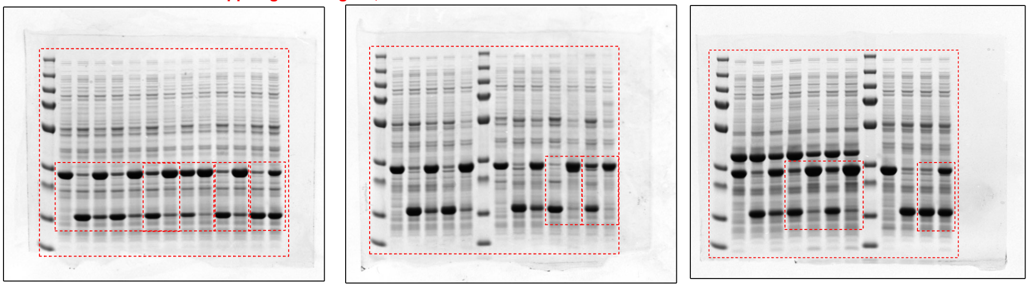

red boxes are shown as a cropped gels in Fig. 3g and Extended Fig. 3a

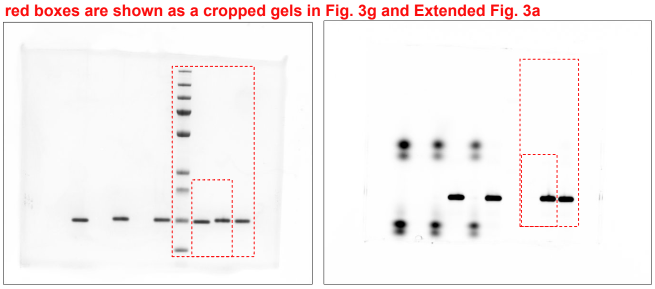

red boxes are shows as a cropped western blot in Fig. 3h and extended Fig. 3b

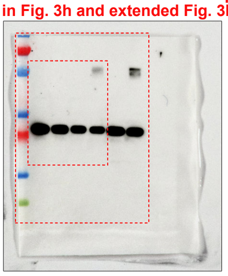

red boxes are shown as a cropped gels in Fig. 3i and Extended Fig. 4c

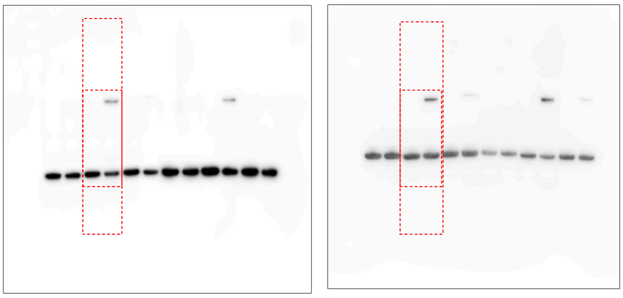

red boxes are shown as cropped gels in Fig. 4a,e and S9b

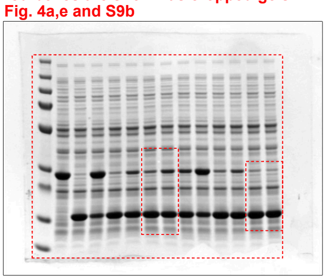

red boxes are shown as cropped gels in Fig. 4e and S9b

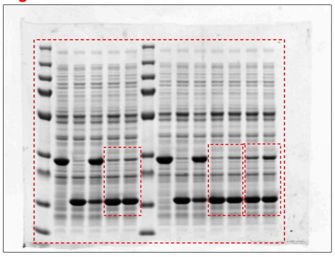

red boxes are shown as cropped gels in Fig. 4e and S9b

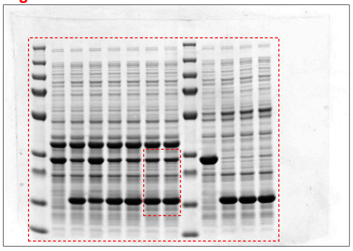

red boxes are shown as cropped gels in Fig. 4a,e and S9c

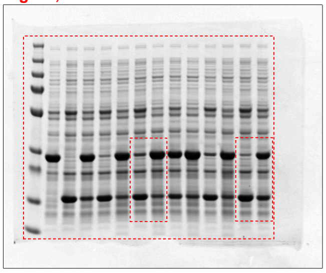

red boxes are shown as cropped gels in Fig. 4e and S9c

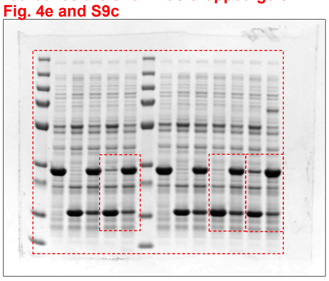

red boxes are shown as cropped gels in Fig. 4e and S9c

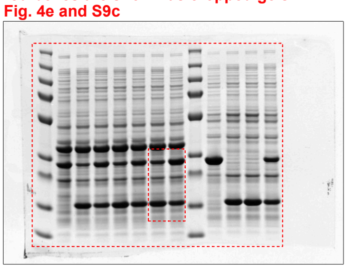

red boxes are shown as cropped gels in Fig. 5b and Extended Fig. 7b

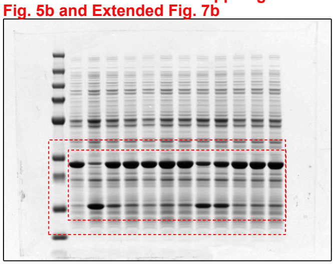

red boxes are shown as cropped gels in Fig. 5b and Extended Fig. 7b

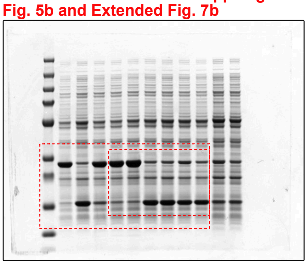

red boxes are shown as cropped gels in Fig. 5b and Extended Fig. 7c

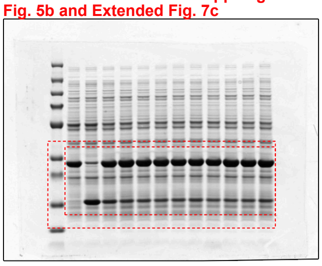

red boxes are shown as cropped gels in Fig. 5b and Extended Fig. 7c

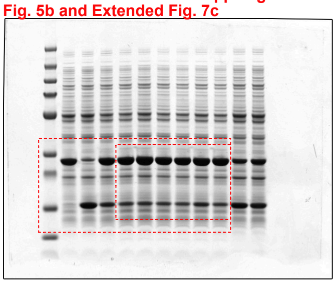

red boxes are shown as cropped gels in Fig. 5e and Extended Fig. 8a

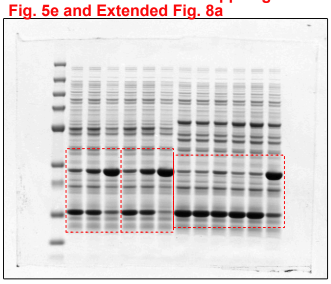

red box is shown as a cropped gel in Fig. 5f

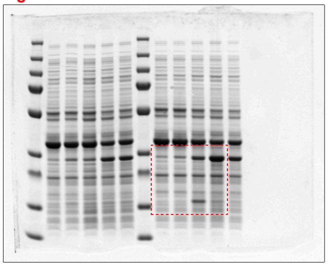

red box is shown as a cropped western blot in Extended Data Fig. 3b

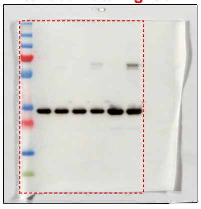

red box is shown as a cropped Western blot in Extended Data Fig. 4a

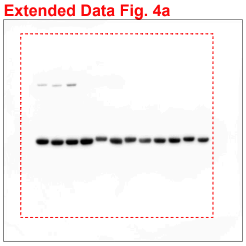

red box is shown as a cropped gel in Extended Data Fig. 4a

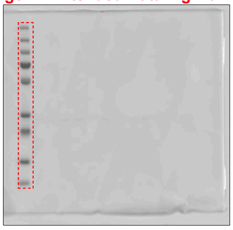

red box is shown as a cropped gel in Extended Data Fig. 4b

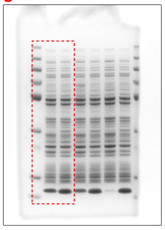

red box is shown as a cropped western blot in Extended Data Fig. 4b

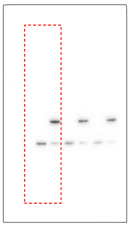

red box is shown as a cropped gel in Extended Data Fig. 4b

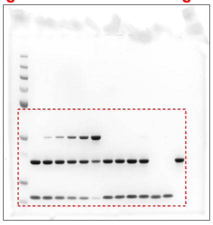

red boxs are shown as cropped gels in Extended Data Fig. 4c

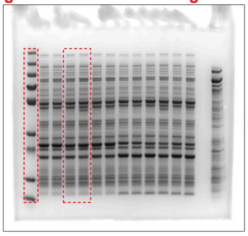

red box is shown as a cropped gel in Fig. Extended Data Fig. 4c

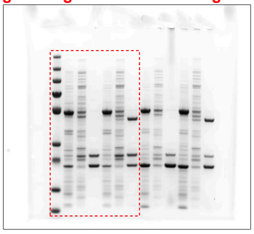

red box is shown as a cropped gel in Fig. Extended Data Fig. 4c

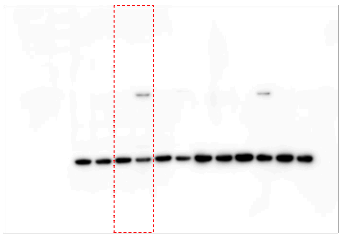

red box is shown as a cropped gel in Fig. Extended Data Fig. 4c

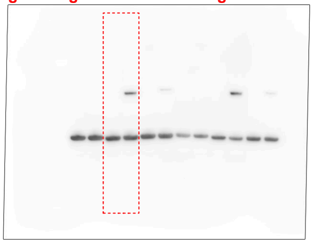

red box is shown as a cropped gel in Extended Data Fig. 6a

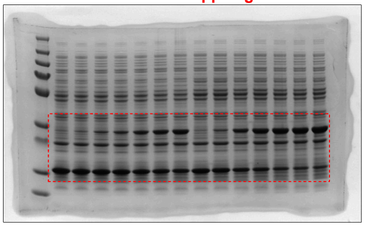

red boxes are shown as cropped gels in Extended Data Fig. 6b and Fig S15

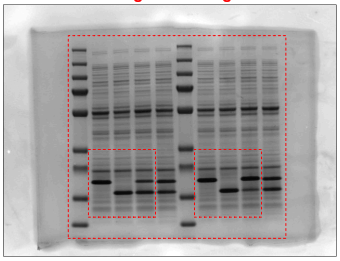

red boxes are shown as cropped gels in Extended Data Fig. 6b and Fig S15

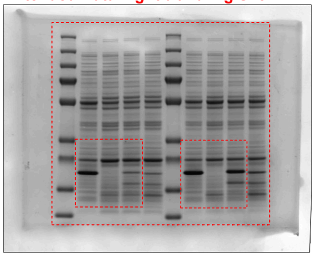

red boxes are shown as cropped gels in Extended Data Fig. 6b and Fig S15

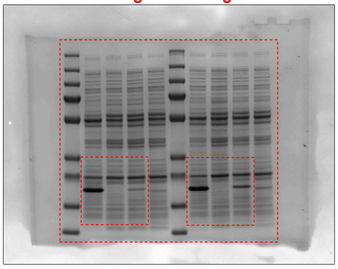

red boxes are shown as cropped gels in Extended Data Fig. 6c

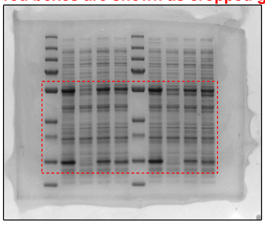

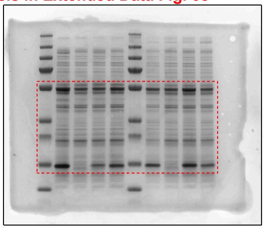

red boxes are shown as cropped gels in Extended Data Fig. 6e and Fig. S12

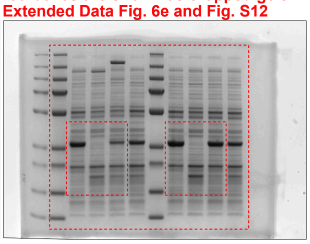

red boxes are shown as cropped gels in Extended Data Fig. 6e and Fig. S12

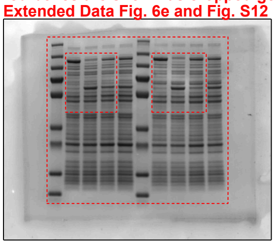

red boxes are shown as cropped gels in Extended Data Fig. 6e and Fig. S12

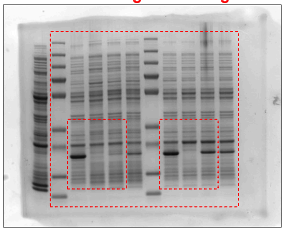

red boxes are shown as cropped gels in Extended Data Fig. 6e and Fig. S13

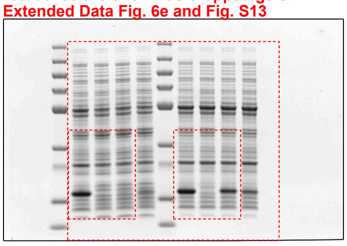

red boxes are shown as cropped gels in Extended Data Fig. 6f and Fig. S13

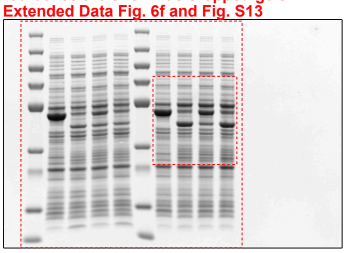

red boxes are shown as cropped gels in Extended Data Fig. 6f and Fig. S13

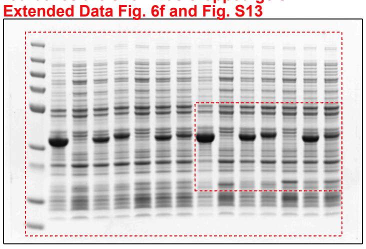

red boxes are shown as cropped gels in Extended Data Fig. 7

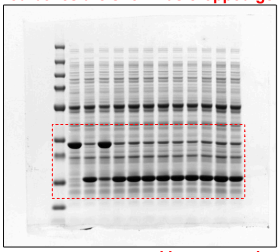

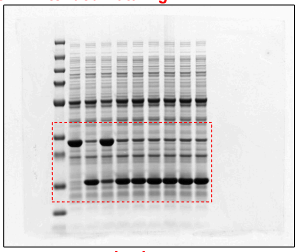

red boxes are shown as cropped gels in Extended Data Fig. 7

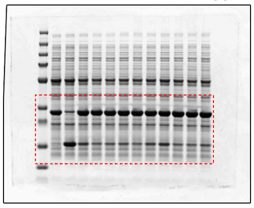

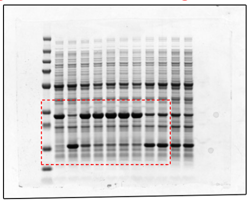

red boxes are shown as a cropped gels in Extended Data Fig. 8b

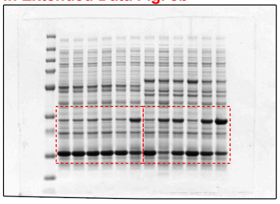

red box is shown as a cropped gel in Extended Data Fig. 8c

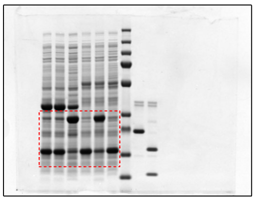

red box is shown as cropped gel in Extended Data Fig. 8c

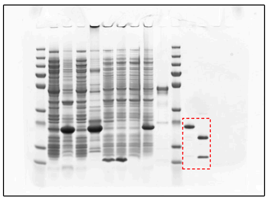

red box is shown as a cropped gel in Fig. S1b

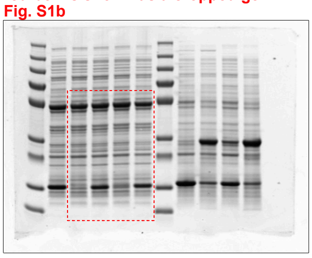

red box is shown as a cropped gel in Fig. S1b

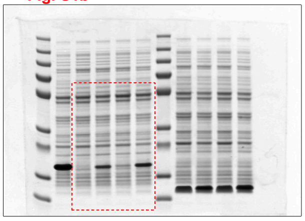

red box is shown as a cropped gel in Fig. S1b

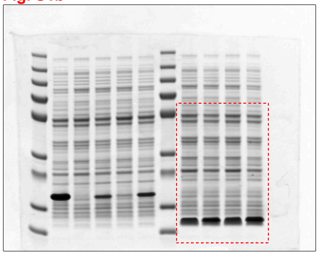

red box is shown as a cropped gel in Fig. S2b

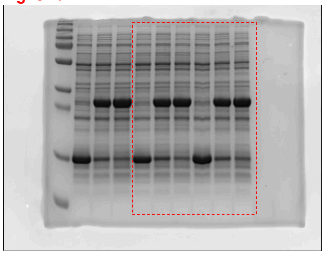

red box is shown as a cropped gel in Fig. S2b

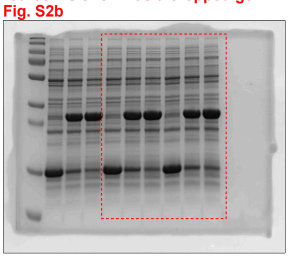

red box is shown as a cropped gel in Fig. S2b

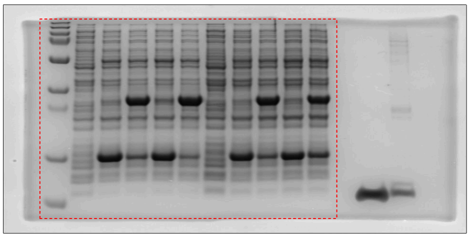

red boxes a shows as a cropped gels in Fig. S2c

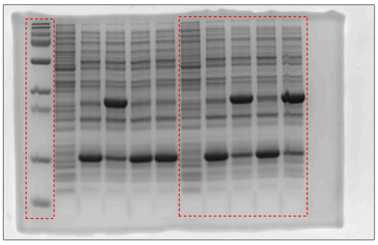

red box is shown as a cropped gel in Fig. S2c

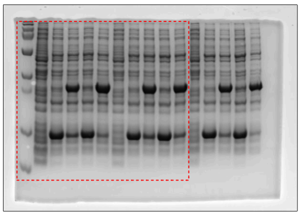

red box is shown as a cropped gel in Fig. S3a

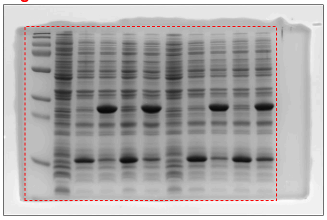

red box is shown as a cropped gel in Fig. S3a

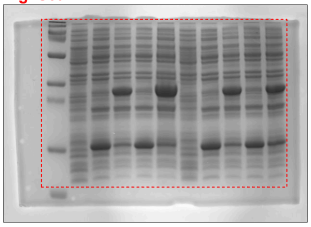

red boxes are shown as cropped gels in Fig. S3a

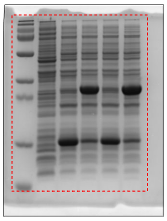

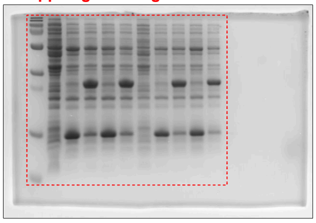

red box is shown as a cropped gel in Fig. S3a

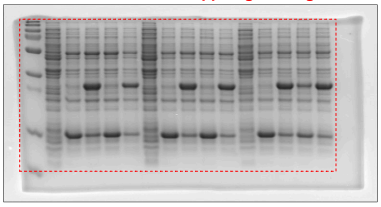

red box is shown as a cropped gel in Fig. S3b

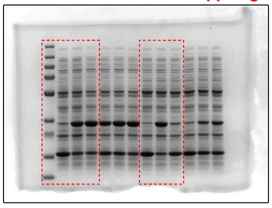

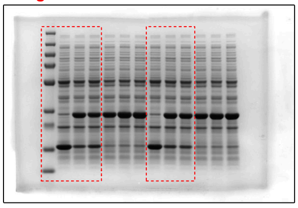

red box is shown as a cropped gel in Fig. S3b

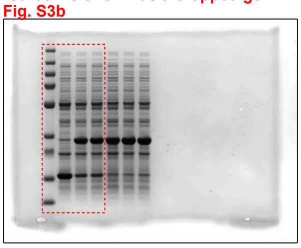

red box is shown as a cropped gel in Fig. S3c

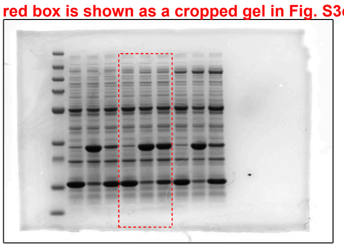

red box is shown as a cropped gel in Fig. S4a

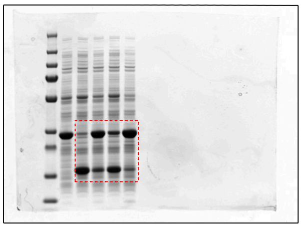

red boxes are shown as cropped gels in Fig. S4b

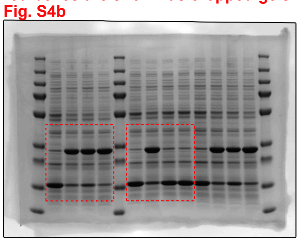

red boxes are shown as cropped gels in Fig. S4d

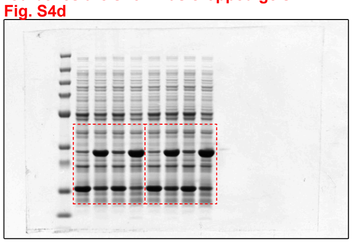

red boxes are shown as cropped gels in Fig. S4d

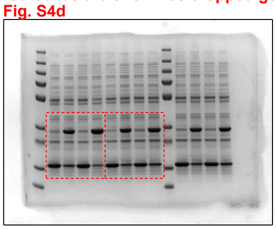

red box is shown as a cropped gel in Fig. S4d

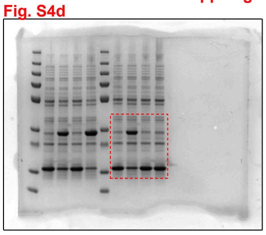

red boxes are shown as cropped gels in Fig. S4d

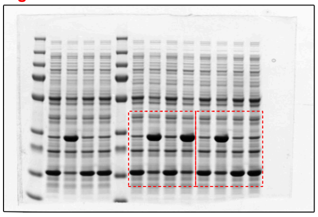

red box is shown as a cropped gel in Fig. S4d

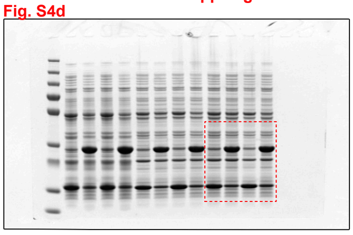

red boxes are shown as cropped gels in Fig. S7

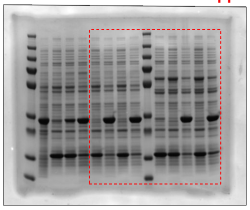

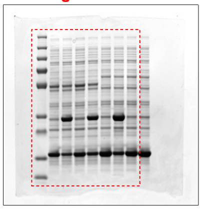

red boxes are shown as cropped gels in Fig. S12

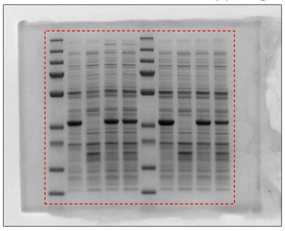

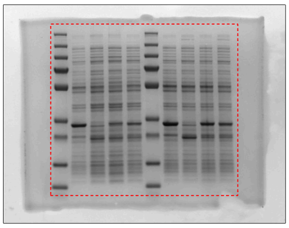

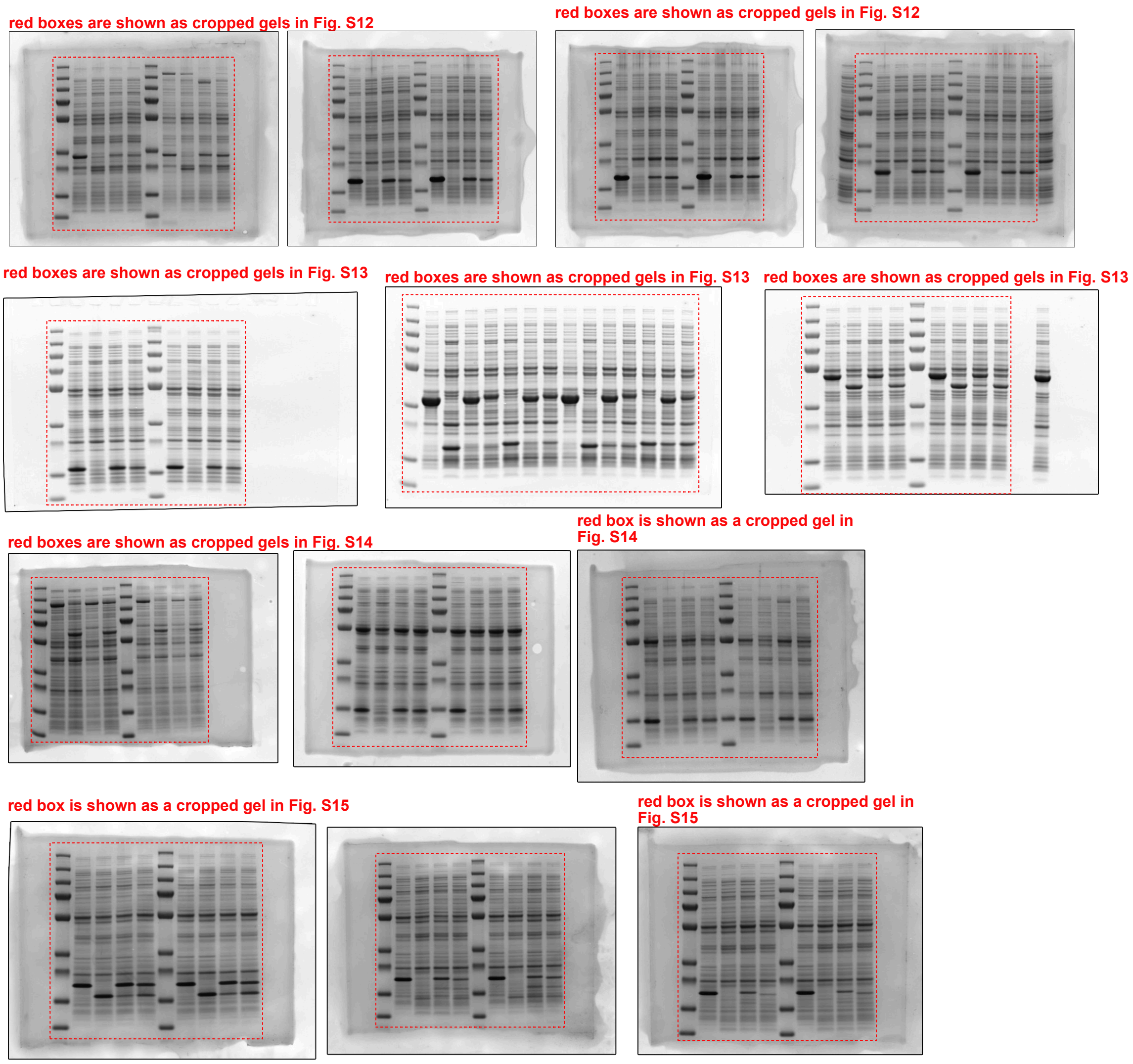

Supplementary Figure 21: Fully uncropped and unprocessed gels.

Supplementary methods

General methods: Plasmids and Reagents

Genes encoding GST-E51TAG, tyrosinase, IL-2, RanGAP, hGH and Histone H3 mutants were ordered as DNA strings from Twist biosciences and cloned into the pBAD vector via standard restriction cloning with enzymes from New England Biolabs. Single point mutants of OppA as well as insertion of a 3C site on UbK63 were done introduced via Site-directed, Ligase-Independent Mutagenesis (SLIM)[5]. Recombination plasmid pSIJ8, pSIMcpf1 and, pTF-lacZ were purchased from Addgene (Addgene ID: 68122, #153034, #153036). Oligonucleotide primers were ordered from Microsynth AG unless otherwise specified. General solvents and chemical reagents were purchased from Sigma Aldrich, cslabs, Fisher Scientific, Carbolution or Acros Organics. Fmoc building blocks of Photoleucine, Propagylglycine and Chloroalanine as well as Boc protected Lipoyllysine, Dap(Alloc), 3-pyridylalanine and, Biotinyllysine, were purchased from Iris Biotech GmbH. All reagents were used without further purification.

SDS-PAGE gels (Bolt™ Bis-Tris Plus Mini Protein Gels, 4-12%, Invitrogen) were run on a Bolt™ Mini Gel Tank (Invitrogen) system (165 V for 40 minutes) and stained with Quick Coomassie Stain (Generon). As a marker, PageRuler Prestained Plus Protein Ladder 10-250 kDa (ThermoFisher) was used. Proteins were transferred onto a nitrocellulose membrane using a Bio-Rad Trans-blot Turbo Transfer System. After transfer, the membrane was blocked with 5% skim milk in TBS-T buffer for 1 h at RT and incubated with the appropriate HRP-coupled antibody. Imaging of western blots was performed on an Amersham ImageQuant™ 800 using Immobilon® Forte Western HRP Substrate (Millipore).

Peptide and protein LC-MS was performed on an Agilent Technologies 1260 Infinity LC-MS system with a 6310 Quadrupole spectrometer. Proteins were measured on a Phenomenex Jupiter C4 300 A LC Column (150 x 2 mm, 5 μm) and peptides, on a Luna Omega PS C18 (100 x 2.1 mm, 3 μm). The solvent system consisted of 0.1% formic acid in water (solvent A) and 0.1% formic acid in acetonitrile (solvent B). Both proteins and peptides were analyzed in positive mode using OpenLab Chemstation (Agilent, LTS01.11 (251)).

Synthesis of peptides via solid phase peptide synthesis

All peptides were synthesized via solid phase peptide synthesis (SPPS). Reactions were performed in plastic syringes with a frit using <1g of resin. SPPS was done using the Fmoc-strategy using a 2-Chlorotrityl chloride resin. For charging, 2 eq. of Fmoc protected amino acid (Boc-Lys(Fmoc)-OH) and 3 eq of DIPEA were dissolved in DCM and added to 1 eq of resin (1.5 mmol/g maximum capacity, 100-200 mesh) and incubated for 1 h on a roller at RT. Resin was then washed 5 times with DCM and 5 times with DMF. Fmoc deprotection was performed by adding a 20% piperidine (v/v) solution in DMF and incubated for 10 min at RT. This process was performed twice for complete deprotection followed by washing the resin 5 times with DMF. For subsequent coupling of amino acids, a coupling solution was prepared by dissolving 2 eq. of Fmoc protected amino acid (Fmoc-X-OH), 1.8 eq. HATU and 3 eq. DIPEA in DMF and mixing for 5 min at RT. Coupling solution was added to the resin and the mixture incubated on a roller for 1 h at RT. This process was repeated depending on the length of peptide being synthesized. For tripeptides a third coupling step was done with a Boc protected amino acid (Boc-Z-OH).

For cleavage of the protected peptide off the resin, the resin was washed 5 times with DMF and 5 times with DCM and cleavage was performed by adding 20% HFIP in DCM (v/v) for 10 minutes at RT. This process was repeated 2 times, each time collecting the filtrate. Solvent from the filtrate was evaporated under reduced pressure.
Deprotection was performed by adding 95% TFA with 2.5% water and 2.5% TIPS and stirred at RT till deprotection was complete, monitoring the reaction via LC-MS. Deprotected peptide was subsequently precipitated in cold ether and dissolved in water. Any remaining ether was evaporated under low pressure and the resulting peptide solution was flash frozen in liquid nitrogen and lyophilized to obtain a dry powder. Purity was judged by HPLC-MS to be greater than 95%.

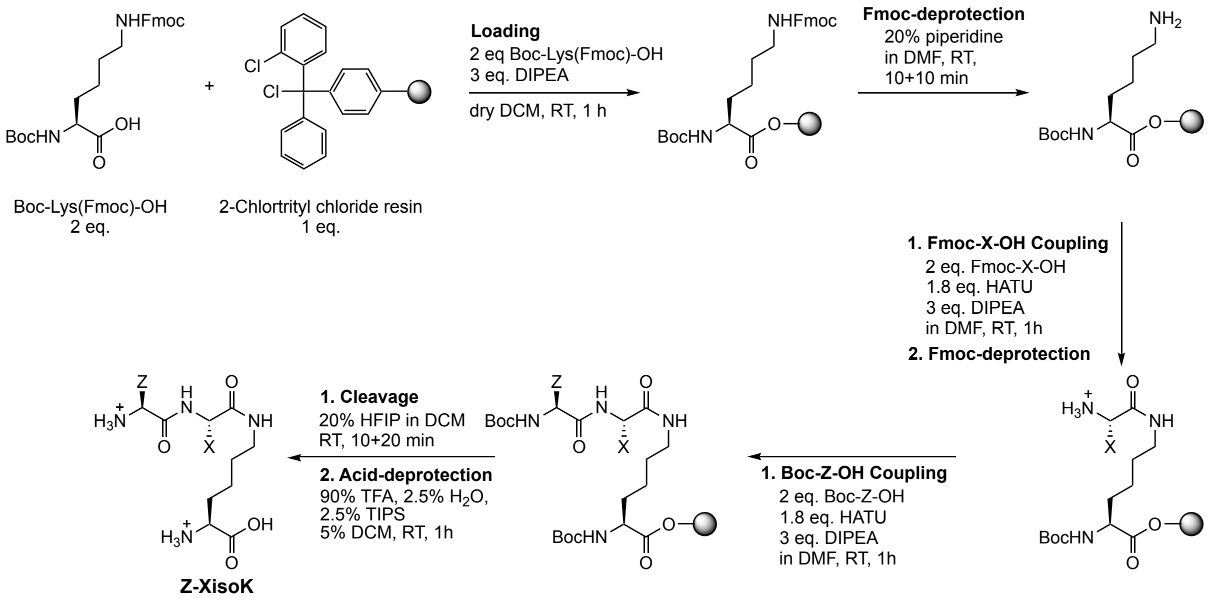

Scheme S1: Fmoc SPPS strategy used for synthesis of peptides in this study.

Expression and purification of OppA and variants

Chemically competent *E. coli* K12 Δ*Opp*A cells were transformed with pBAD_OppA_His6. After recovery in 1 mL SOC for 1 h at 37 °C, the cells were cultured in 50 mL of 2-YT supplemented with 100 µg/mL Ampicillin overnight at 37 °C. The overnight culture was then diluted to OD_{600} 0.05 in 2-YT supplemented with 50 µg/mL Ampicillin and grown at 37 °C, 200 rpm till OD_{600} 0.6. Protein expression was induced with 0.05% L-arabinose and the culture grown for 3 h at 37 °C, 200 rpm. Cells were harvested by centrifugation at 7000 x g for 20 min and pellets flash frozen in liquid nitrogen and stored at -20 °C.

Cell pellets were resuspended in lysis buffer (20 mM Tris-HCl pH 8, 300 mM NaCl, 30 mM imidazole, 1 mM PMSF) (30 ml/liter culture) and cells lysed using a cell disruptor (CF1 Constant Systems Ltd., 40 Kpsi). Lysed cells were centrifuged at 14,000 x g, 20 minutes and cleared lysate loaded on a 5ml HisTrap HP column (Cytiva) pre-equilibrated with wash buffer (20 mM Tris-HCl pH 8, 300 mM NaCl, 30 mM imidazole). The loaded column was then washed with 5 column volumes (CV) of wash buffer. Protein was eluted with a gradient from 30 to 300 mM imidazole over 20 CV, collecting 1.4 ml fractions. Fractions containing protein were pooled and concentrated using Amicon® centrifugal filter units (Millipore, 30 kDa MWCO) and then injected on a HiLoad 16/600 Superdex 75 pg size exclusion (SE) column (Cytiva) equilibrated with SE buffer (2x PBS pH 7). SE fractions containing protein were then pooled and concentrated. Any endogenously bound peptides were removed by partially unfolding OppA with 3 M Guanidine hydrochloride (GdnHCl) in SE buffer and removing the peptides via dialysis (Pur-A-Lyzer™ Maxi, 12 kDa MWCO, Sigma-Aldrich) in SE buffer containing decreasing amounts of GdnHCl from 3 M to 0 M. Resulting protein was then flash frozen in liquid nitrogen and stored at -80 °C till further use.

For crystallization of wt-OppA, the protein was partially unfolded as described above in 20 mM Tris-HCl pH 7.5 100 mM NaCl, 3M GdnHCl. During dialysis, 0.5 mM G-SisoK was added to the buffer (20 mM Tris-HCl pH 7.5 100 mM NaCl) to facilitate folding around G-SisoK.

Crystallization and structure determination of the OppA:G-SisoK complex

Suitable crystallization conditions were identified using commercially available screens with purified OppA at a concentration of 47 mg/mL in 20 mM Tris-HCl pH 7.5, 100 mM NaCl and 10 mM G-SisoK. Crystals grew after 2 days at 20 °C in a 2:1 drop ratio of protein solution to crystallization solution (15% EtOH, 40 % v/v Pentaerythritol propoxylate).

X-ray diffraction data for the OppA:G-SisoK complex were collected at beamline P13, operated by EMBL Hamburg at the PETRA III storage ring (DESY, Hamburg, Germany). Diffraction images were processed using the XDS software suite[6] (**Table 5**), and ligand topology parameters were generated with PRODRG. Phases were determined by Patterson search methods applying PHASER 2.7.0[7] using the previously solved structure of *Escherichia coli* OppA (PDB ID 3TCF[8]) as a model. Structure refinement was performed with REFMAC 5[9] followed by iterative model building in COOT (v. 0.9).[10] Water molecules were incorporated using ARP/wARP 8.0[11], and final model validation was performed with MOLPROBITY 4.0.4[12]. The refined OppA:G-SisoK structure has been deposited in the RCSB Protein Data Bank under the accession code PDB ID: 9RD1 (resolution: 2.5 Å, R_{work} = 22.9%, R_{free} = 25.9%; **Table 5**).

Expression and purification of Tyrosinase

Chemically competent *E. coli* BL21 (DE3) were transformed with pET28_Tyrosinase_H6 (encoding for tyrosinase from *Priesta megaterium*). After recovery with 1 mL of SOC medium for 1 h at 37 °C, the cells were cultured overnight in 50 mL 2-YT medium containing kanamycin (50 µg/mL) at 37 °C, 200 rpm. The overnight culture was diluted to an OD_{600} of 0.05 in 1 L of fresh 2-YT medium supplemented with kanamycin (25 µg/mL) and cultured at 37 °C with shaking (200 rpm) until OD_{600} reached 0.5.

IPTG was added to a final concentration of 0.4 mM and protein expression was induced for 18 h at 37 °C. The cells were harvested by centrifugation (4000 × g, 20 min, 4 °C), resuspended in lysis buffer (20 mM Tris-HCl pH 8.0, 300 mM NaCl, 30 mM imidazole, 0.2 mM PMSF) and lysed via sonication in an ice water bath. The lysed cells were centrifuged (15,000 × g, 40 min, 4 °C) and the cleared lysate was applied to Ni Sepharose fast flow beads (1mL slurry/ 1L culture) pre-equilibrated with Ni-NTA wash buffer (20 mM Tris-HCl pH 8.0, 300 mM NaCl, 30 mM imidazole) and incubated on a roller for 1 h at 4 °C. After incubation, the mixture was transferred to an empty plastic column and washed with 10 CV of wash buffer (20 mM Tris-HCl pH 8.0, 300 mM NaCl, 30 mM imidazole). Tyrosinase was eluted in 1 mL fractions with wash buffer supplemented with 300 mM imidazole and 0.02 mM $CuSO_4$. The fractions containing the protein were pooled and buffer was exchanged to PBS pH 7.4 supplemented with 15 % glycerol and 0.02 mM $CuSO_4$ using an Amicon® centrifugal filter units with an appropriate MWCO (Millipore). Protein concentration was calculated from the measured A280 absorption (extinction coefficients were calculated with ProtParam (https://web.expasy.org/protparam/). Tyrosinase was flash frozen using liquid nitrogen and stored at -80 °C until further use.

Expression and purification of eGFP nanobodies and their yield determination

In order to determine yields of amber suppressed eGFPNb, expression and purification were performed as with other XisoK bearing proteins with a few modifications. Both *E. coli* K12 and isoK12 were grown in 25 mL 2-YT cultures manually inducing expression with 0.05% arabinose at an OD_{600} of 0.6. 300 uL of Ni-NTA bead slurry was used for each purification and after loading the beads with lysate an additional high salt wash was done with wash buffer containing 1 M NaCl to remove non-specifically bound proteins. Protein was eluted in elution buffer until no more protein was detected in the flow-through, monitoring with Bradford's reagent. Eluted protein was buffer exchanged in 1xPBS pH 7, 5 times and concentrations determined using a NanoPhotometer® NP60 (Implen). To check protein purity as well as if all the protein bound to the beads, eluted protein and lysate flow-though were analyzed via SDS-PAGE.

Evolution of HisoKRS

Electrocompetent *E. coli* DH10β cells containing a positive selection plasmid pPylT_CAT111TAG were transformed with a pBK_*Mb*PylRS_lib (MbPylRS library with positions A276, Y271, L274, C313 and M315 randomized using degenerate NNK primers). After recovery with 5 mL SOC for 1 hour at 37 °C. cells were added to 100 mL of 2-YT supplemented with Ampicillin (100 µg/mL) and Tetracycline (17 µg/ml) and grown to an OD_{600} of 0.6. Cells were then plated in a dilution series on 24 cm autoinduction agar plates containing 0.5 mM G-HisoK and incubated overnight at 37 °C. Plates with distinct single colonies were scraped and the plasmids were isolated from the cell mass and the pBK_*Mb*PylRS_lib was isolated on a 1% agarose gel. Electrocompetent *E. coli* DH10β cells containing a negative selection plasmid pYOBB_Barnase3TAG were then transformed with the isolated library and after recovery with 1 mL SOB for 1 h at 37 °C, cells were added to 100 ml of 2-YT

supplemented with Ampicillin (100 µg/mL) and Chloramphenicol (50 µg/mL) and grown to an OD_{600} of 0.6. For negative selection, cells were then plated on LB-agar plates supplemented with Ampicillin (100 µg/mL), Chloramphenicol (50 µg/mL) and 0.2% arabinose and grown overnight at 37 °C. Plates were scraped and library plasmid isolated for a final positive selection round using a pPylT_GFP_N150TAG_CAT111TAG double reporter plasmid. Single colonies were then picked into a 96 deep well plate containing 1 mL non-inducing media/well and grown overnight at 37 °C. Two 96 deep well plates containing autoinducing media[13] with and without 0.5 mM G-HisoK were inoculated with the overnight culture and grown for 24 h. sfGFP fluorescence was measured for both plates on a Varioscan Lux plate reader (Thermo Scientific). Clones with sufficient fluorescence above negative sample were sent for Sanger sequencing.

On bead CuAAC labeling of eGFP-NB with Picolyl-Azide-Sulfo-Cy5

eGFP-NB bearing either BocK, XisoK (Prg) or PraK[4] at position R75 was expressed and purified as described in '**Expression of XisoK bearing proteins**' and '**Purification of His6 tagged proteins**'. Purified proteins were buffer exchanged into PBS pH 7 using Amicon® centrifugal filter units (Millipore, 3 kDa MWCO), and 20 µM of each eGFP-NB variant was bound to magnetic Ni-NTA beads (Cube biotech) . Beads were washed 2 times with PBS pH 7 and then incubated with CuAAC mix containing 100 µM AF647-Picolyl-Azide (Jena Bioscience), 50 µM $CuSO_4$, 250 µM THPTA and 2.5 mM ascorbic acid in PBS pH 7 for 30 minutes at room temperature. Beads were then washed 5 times with PBS pH 7 and protein was eluted with 300 mM imidazole in PBS pH . Eluted protein and flow-through samples were run on an SDS-PAGE and labeling visualized via in-gel fluorescence (iBright™ FL1500, ThermoFisher Scientific).

Photocrosslinking of diazirine bearing proteins in cells

Chemically competent *E. coli* K12 cells were co-transformed with pBAD_POI_His6 (either sfGFP or GST encoding a protein of interest with a C-terminal His6 tag and a TAG codon at the indicated position) and pEVOL_*Ma*PylRS_IP (encoding two copies of *Ma*PylRS H227I, Y228P and *Ma* $tRNA_{CUA}$) for incorporating pLisoK or pEVOL_DiazKRS (encoding two copies of DiazKRS[14] and *Mm* $tRNA_{CUA}$). After recovery in 1 mL SOC for 1 h at 37 °C, cells were added to 5 mL of 2-YT supplemented with Ampicillin (100 µg/mL) and Chloramphenicol (50 µg/mL) and grown overnight at 37 °C. Autoinducing media[13] supplemented with Ampicillin (100 µg/mL) and Chloramphenicol (50 µg/mL) and containing either no, 2mM BocK or 2mM G-pLisoK were inoculated with the overnight culture and grown at 37 °C overnight. The resulting culture was diluted to an OD_{600} of 1 and +UV samples were irradiated with UV light (15 W, 365 nm) using a lamp (Vilber, VL-215.L) for 15 minutes. +UV and -UV samples were run on an SDS-PAGE and analyzed via western blot using an anti-His peroxidase-coupled antibody (Sigma).

Tyrosinase-mediated labelling of PisoK bearing proteins

20 µM of protein (either Ub wt, UbK63PisoK or 3C-UbK63PisoK) was incubated with 160 µM p-cresol and 0.4 µM tyrosinase in tyrosinase buffer (20 mM potassium phosphate buffer pH 6.5, 100 mM NaCl) and incubated at room temperature for 2 hours. Reaction was quenched with 1 mM TCEP and 1 mM BCN-OH for 10 minutes. Cleavage of the 3C-UbK63PisoK construct N-terminus was done with HRV 3C-protease (ThermoFischer) to obtain single-labeled protein.

Chemical crosslinking of protein-protein complexes using ClAisoK in living *E. coli*

Affibody-ProteinZ:

Chemically competent *E. coli* K12 cells were co-transformed with pBAD_Affibody_D36TAG_His6, pBAD_RSF1035_Strep_SUMO_ProteinZ_N7C and pEVOL_*Mb*PylRS_C313V (encoding two copies of *Mb* PylRS with a C313V mutation and *Mm* $tRNA_{CUA}$) or pEVOL_wt_*Mb*PylRS. After recovery in 1 mL SOC for 1 h at 37 °C, cells were added to 5 mL of 2-YT supplemented with Ampicillin (100 µg/mL), Chloramphenicol (50 µg/mL) and Kanamycin (50 µg/mL) and grown overnight at 37 °C, 200 rpm. Autoinducing[13] (2 mL cultures in a 24-well plate) supplemented with Ampicillin (100 µg/mL), Chloramphenicol (50 µg/mL) and Kanamycin (50 µg/mL) and containing either no ncAA, 2 mM G-ClAisoK or 2 mM G-AisoK were inoculated with the overnight culture and grown at 37 °C, 200 rpm overnight.
After overnight incubation, OD_{600} was measured, samples were normalized and subjected to SDS-PAGE followed by either Coomassie staining or western blot analysis using an anti-His peroxidase-coupled antibody (Sigma) or antiStrep-HRP (StrepMAB-Classic HRP, IBA, Cat.No. 2-1509-001).

sfGFP dimer:

Chemically competent *E. coli* K12 cells were co-transformed with pBAD_sfGFP_N150TAG_His6 (and mutants E173C, E173H, E173A or V207K) and pEVOL_*Mb*PylRS_C313V (encoding two copies of *Mb* PylRS with a C313V mutation and *Mm* $tRNA_{CUA}$) or pEVOL_wt_*Mb*PylRS. Expression and downstream analysis were performed analogously to the above-described procedure for Affibody-ProteinZ.

Rab1b-DrrA

Chemically competent *E. coli* K12 cells were co-transformed with pBAD_Rab1b_R79TAG_His6_RBS_Strep_TEV_DrrA(339-522)_D512C (encoding both target genes in a polycistronical manner) and pEVOL_*Mb*PylRS_C313V (encoding two copies of *Mb* PylRS with a C313V mutation and *Mm* $tRNA_{CUA}$) or pEVOL_wt_*Mb*PylRS. Expression and downstream analysis were performed analogously to the above-described procedure for Affibody-ProteinZ. Ni-NTA purification was performed as described in **"Purification of His6 tagged proteins"**.

Chemical crosslinking of Affibody and ProteinZ in vitro

Affibody bearing either AisoK or ClAisoK at position 36 and a SUMO-ProteinZ N7C mutant were expressed and purified as described in sections "Expression of XisoK bearing proteins" and **"Purification of His6 tagged proteins"**. For in vitro crosslinking assays Affibody variants and the SUMO-ProteinZ N7C mutant were diluted to 50 µM each into PBS pH 7.4 supplemented with 1 mM TCEP and incubated at 37 °C. At denoted time points, 2 µL samples were taken and quenched by the addition of 8 µL 4× SDS Laemmli buffer. After boiling at 95 °C for 10 min, the samples were loaded on SDS–PAGE and visualized by Coomassie staining.

Determination of doubling times of isoK12 and K12 in AI and 2-YT media

Single colonies of *E. coli* K12 and *iso*K12 were cultured in 20 ml of 2-YT and grown to an OD_{600} of 0.6 at 37 °C, 200 rpm. These cultures were then diluted to an OD_{600} of 0.05 in pre-warmed 2-YT or autoinducing media[13] and cultured at 37 °C, 200 rpm while monitoring OD_{600}

at regular intervals. To determine doubling times the slope of time (minutes) vs log2(OD_{600}) at the exponential phase was calculated. Analysis was done using GraphPad Prism Version 10.

Platereader based sfGFP fluorescence measurements

sfGFP fluorescence time courses of *E. coli* cultures were measured on a Tecan Spark® Multimode Microplate Reader with a humidity cassette in order to prevent evaporation. *E. coli* cultures containing the appropriate plasmids were diluted to an OD_{600} of 0.05 in the relevant media and grown to an OD_{600} of 0.6 and sfGFP expression was induced with 0.05% arabinose and ncAA added to the media. 200 uL of this culture was added to the well of a 96-well microplate with a clear bottom (Greiner Bio-One µClear™ Cat.no. 655096) and the plate sealed with a breathe-EASIER (Diversified Biotech) membrane. sfGFP fluorescence was measured from the bottom every 10 minutes while shaking in-between reads at 37 °C.

Table 1: Plasmids used in this study

**Plasmid**	**Resistance**	**Description**
pBAD_sfGFP_N150TAG_H6	Ampicillin	sfGFP-N150TAG with a C-terminal His6 tag under an arabinose promoter
pBAD_sfGFP_wt_H6	Ampicillin	sfGFP wt with a C-terminal His6 tag under an arabinose promoter
pBAD_Ub_K63TAG_H6	Ampicillin	Ubiquitin-K63TAG with a C-terminal His6 tag under an arabinose promoter
pEVOL_*Mb*PylRS	Chloramphenicol	Two copies of *Mb*PylRS wt under an arabinose promoter and a glnS promoter and *Mm*$tRNA_{CUA}$ under a proK promoter.
pEVOL_*Mb*PylRS_C313V	Chloramphenicol	Two copies of *Mb*PylRS C313V under an arabinose promoter and a glnS promoter and *Mm*$tRNA_{CUA}$

		under a proK promoter
pEVOL_*Ma*PylRS_IP	Chloramphenicol	Two copies of *Ma*PylRS H227I, Y228P under an arabinose promoter and a glnS promoter and *Ma*tRNA$_{CUA}$ under a proK promoter
pEVOL_HisoKRS	Chloramphenicol	Two copies of *Mb*PylRS Y271L C313T under an arabinose promoter and a glnS promoter and *Mm*tRNA$_{CUA}$ under a proK promoter
pEVOL_DiazKRS	Chloramphenicol	Two copies of DiazKRS under an arabinose promoter and a glnS promoter and *Mm*tRNA$_{CUA}$ under a proK promoter
pEVOL_MbPylRS_Y271A_L274M	Chloramphenicol	Two copies of *Mb*PylRS Y271A L274M under an arabinose promoter and a glnS promoter and *Mm*tRNA$_{CUA}$ under a proK promoter
pEVOL_MmPylRS_AF	Chloramphenicol	Two copies of *Mm*PylRS Y308A Y384F under an arabinose promoter and a glnS promoter and *Mm*tRNA$_{CUA}$ under a proK promoter
pBAD_eGFP-NB_wt_H6	Ampicillin	eGFP nanobody wt with a C-

		terminal His6 tag under an arabinose promoter
pBAD_eGFP-NB_R17TAG_H6	Ampicillin	eGFP nanobody-R17TAG with a C-terminal His6 tag under an arabinose promoter
pBAD_eGFP-NB_R75TAG_H6	Ampicillin	eGFP nanobody-R75TAG with a C-terminal His6 tag under an arabinose promoter
pBAD_GST_E51TAG_H6	Ampicillin	GST-E51TAG with a C-terminal His6 tag under an arabinose promoter
pBAD_3C_Ub_K63TAG_H6	Ampicillin	Ub-K63TAG with a N-terminal 3C cleavage site and a C-terminal His6 tag under an arabinose promoter
pPylT_CAT_111TAG	Tetracycline	Chloramphenicol acetyl transferase with a TAG stop codon at position 111
pBK_*Mb*PylRS_lib	Ampicillin	*Mb*PylRS library with positions A276, Y271, L274, C313 and M315 randomized with NNK primers under a *gln*S promoter
pYOBB_Barnase_3TAG	Chloramphenicol	Barnase with a TAG stop codon at position 3
pPylT_sfGFP150TAG_CAT_111TAG	Tetracycline	sfGFP with a TAG stop codon at position 150 under an

		arabinose promoter and Chloramphenicol acetyl transferase with a TAG stop codon at position 111
pEVOL_ *Mb*PylRS_*pep*A	Chloramphenicol	*Mb*PylRS under an arabinose promotoer and pepA under a *gln*S promoter and *Mm*tRNA$_{CUA}$ under a proK promoter
pEVOL_ *Mb*PylRS_*pep*N	Chloramphenicol	*Mb*PylRS under an arabinose promotoer and pepN under a *gln*S promoter and *Mm*tRNA$_{CUA}$ under a proK promoter
pEVOL_*Mb*PylRS_*opp*A_wt	Chloramphenicol	*Mb*PylRS under an arabinose promotoer and *opp*A wt under a *gln*S promoter and *Mm*tRNA$_{CUA}$ under a proK promoter
pEVOL_*Mb*PylRS_*opp*A_D446A	Chloramphenicol	*Mb*PylRS under an arabinose promotoer and *opp*A D446A under a *gln*S promoter and *Mm*tRNA$_{CUA}$ under a proK promoter
pEVOL_*Mb*PylRS_*opp*A_R439A	Chloramphenicol	*Mb*PylRS under an arabinose promotoer and *opp*A R439A under a *gln*S promoter and *Mm*tRNA$_{CUA}$ under a proK promoter

pEVOL_*Mb*PylRS_*opp*A_EP_lib	Chloramphenicol	*Mb*PylRS under an arabinose promotoer and a error prone library of oppA under a glnS promoter and *Mm*tRNA$_{CUA}$ under a proK promoter
pEVOL_*Mb*PylRS_*opp*A_trimer_lib	Chloramphenicol	*Mb*PylRS under an arabinose promotoer and a site saturation library of oppA under a glnS promotoer and *Mm*tRNA$_{CUA}$ under a proK promoter
pEVOL_*Mb*PylRS_*opp*A_iso	Chloramphenicol	*Mb*PylRS under an arabinose promotoer and a site saturation library of oppA-iso under a glnS promoter and *Mm*tRNA$_{CUA}$ under a proK promoter
pEVOL_*Mb*PylRS_*opp*A_Z_lib	Chloramphenicol	*Mb*PylRS under an arabinose promotoer and a site saturation library targeting residues V60, S63, L530, N532 of oppA under a glnS promotoer and *Mm*tRNA$_{CUA}$ under a proK promoter
pEVOL_ *Mb*PylRS_*opp*A_Z1	Chloramphenicol	*Mb*PylRS under an arabinose promotoer and a OppA with mutations V60E, S63G, L530G,

		N532S of oppA under a glnS promotoer and *Mm*tRNA$_{CUA}$ under a proK promoter
pEVOL_ *Mb*PylRS_*opp*A_Z2	Chloramphenicol	*Mb*PylRS under an arabinose promotoer and a OppA with mutations S63A, R439H L530G, N532A of oppA under a glnS promotoer and *Mm*tRNA$_{CUA}$ under a proK promoter
pBAD_oppA_wt_H6	Ampicillin	*opp*A wt with a C-terminal His6 tag under an arabinose promoter
pBAD_oppA_iso_H6	Ampicillin	*opp*A-iso with a C-terminal His6 tag under an arabinose promoter
pBAD_PCNA_wt_H6	Ampicillin	PCNA wt with a C-terminal His6 tag under an arabinose promoter
pBAD_PCNA_K164TAG_H6	Ampicillin	PCNA-K164TAG with a C-terminal His6 tag under an arabinose promoter
pBAD_β-lactamase_wt_H6	Ampicillin	β-lactamase wt with a C-terminal His6 tag under an arabinose promoter
pBAD_β-lactamase_K221TAG_H6	Ampicillin	β-lactamase-K221TAG with a C-terminal His6

		tag under an arabinose promoter
pBAD_SUMO2_wt_H6	Ampicillin	SUMO2 wt with a C-terminal His6 tag under an arabinose promoter
pBAD_SUMO_K45TAG_H6	Ampicillin	SUMO2-K45TAG with a C-terminal His6 tag under an arabinose promoter
pBAD_Calmodulin_wt_strep	Ampicillin	Calmodulin wt with a C-terminal Strep tag under an arabinose promoter
pBAD_Calmodulin_G40TAG_strep	Ampicillin	Calmodulin-G40TAG with a C-terminal Strep tag under an arabinose promoter
pBAD_H3_wt_H6	Ampicillin	Histone H3 wt with a C-terminal His6 tag under an arabinose promoter
pBAD_H3_K122TAG_H6	Ampicillin	Histone H3-K122TAG with a C-terminal His6 tag under an arabinose promoter
pBAD_H3_K79TAG_K122TAG_H6	Ampicillin	Histone H3K79TAG K122TAG with a C-terminal His6 tag under an arabinose promoter
pBAD_H3_K27TAG_ K79TAG_K122TAG_H6	Ampicillin	Histone H3 K27TAG K79TAG K122TAG with a C-terminal His6 tag under an

		arabinose promoter
pBAD_Hsp82_wt_H7	Ampicillin	Hsp82 wt with a C-terminal His7 tag under an arabinose promoter
pBAD_Hsp82_D452TAG_H7	Ampicillin	Hsp82-D452TAG with a C-terminal His7 tag under an arabinose promoter
pBAD_IL-2_wt_H6	Ampicillin	IL-2 wt with a C-terminal His6 tag under an arabinose promoter
pBAD_IL-2_R38TAG_H6	Ampicillin	IL-2 R38TAG with a C-terminal His6 tag under an arabinose promoter
pBAD_TRX_H6_hGH_wt	Ampicillin	hGH with an N-terminal Thioredoxin and His6 tag under an arabinose promoter
pBAD_TRX_H6_hGH_Y35TAG	Ampicillin	hGH Y35TAG with an N-terminal Thioredoxin and His6 tag under an arabinose promoter
pBAD_TRX_H6_hGH_K38TAG	Ampicillin	hGH K38TAG with an N-terminal Thioredoxin and His6 tag under an arabinose promoter
pBAD_GST_TEV_RanGAP1(418-587)_wt_H6	Ampicillin	RanGAP(418-587) with a N-terminal GST tag as well as a TEV protease cut site and a C-terminal His6 tag under an

		arabinose promoter
pBAD_GST_TEV_RanGAP1(418-587)_K524TAG_H6	Ampicillin	RanGAP(418-587) K524TAG with a N-terminal GST tag as well as a TEV protease cut site and a C-terminal His6 tag under an arabinose promoter
pET28_tyrosinase_H6	Kanamycin	Tyrosinase with a C-terminal His6 tag under a T7 promoter
pSIJ8	Ampicillin	Addgene ID: 68122
pEVOL_AcKRS3(TAA)_RBS_MaPylRS_IP(TAG)	Chloramphenicol	AcKRS3 and MaPylRS_IP under a arabinose promoter polycistronically and *Mm*tRNA$_{UUA}$, *Ma*tRNA$_{CUA}$ under separate proK promoters
pBAD_sfGFP_N40TAA_N150TAG_H6	Ampicillin	sfGFP with N40TAA, N150TAG mutations and a C-terminal His6 tag under an arabinose promoter.
pBAD_UbK48TAA_TEV_SUMO2K11TAG_H6	Ampicillin	Ubiquitin with K48TAA mutation followed by a TEV protease cleavage sequenced and SUMO2 with mutation K11TAG and a C-terminal His6 tag under an arabinose promoter.

pSIMcpf1	Hygromycin	Addgene ID#153034
pTF-lacZ	Spectinomycin	Addgene ID#153036
pTF-pepA	Spectinomycin	Donor plasmid with 50 bp upstream and downstream of the *pep*A locus along with a CRISPR array encoding pepA targeting gRNAs
pTF-pepB	Spectinomycin	Donor plasmid with 50 bp upstream and downstream of the *pep*B locus along with a CRISPR array encoding pepB targeting gRNAs
pTF-pepT	Spectinomycin	Donor plasmid with 50 bp upstream and downstream of the *pep*T locus along with a CRISPR array encoding pepT targeting gRNAs
pTF-ypdEF	Spectinomycin	Donor plasmid with 50 bp upstream and downstream of the *ypd*EF locus along with a CRISPR array encoding ypdEF targeting gRNAs
pTF-OppA-Z1	Spectinomycin	Donor plasmid encoding OppA-Z1 with 50 bp upstream and downstream of the oppA locus as well as a CRISPR array encoding

		gRNAs targeting the FRT site present in the Δ*opp*A genome
pTF-OppA-Z2	Spectinomycin	Donor plasmid encoding OppA-Z2 with 50 bp upstream and downstream of the oppA locus as well as a CRISPR array encoding gRNAs targeting the FRT site present in the Δ*opp*A genome

Table 2: Primers used in this study

**Primers**	**Sequence**
OppA_EP_fwd	CGCTTTGAGGAATCCCATATGGGG
OppA_EP_rev	GAAACTGCAGTTAATGGTGATGATGATGGTGC
OppA_D221X_W222X_fwd	CGTAAGAAGACCCTTAAAA**X01X01**GTCGTAAACGAACGAATC
OppA_D221X_W222X_rev	CGTTAGAAGACTTTTAAGGTATAGGCACCG
OppA_R439X_fwd	CGAATGAAGACATGTGGCC**X01**GCAGGCTGGTGTGCTGAC
OppA_R439X_rev	GCATAGAAGACGGCCACATCAAAAGTACCCTGGTGAC
OppA_S460X_fwd	GATATGAAGACTTTCGAAC**X01**TCGATGAATACCGCGC
OppA_S460X_rev	GCTTAGAAGACGTTCGAAAGCATGGTGTTC
OppA_V60X_S63X_fwd	GCTAGAAGACAAAGGT**NNK**CCGGAG**NNK**AATATCAGCCGAGACCT
OppA_V60X_S63X_rev	ATGCGAAGACATACCTTCAATTTTGTGCGG
OppA_L530X_N532X_fwd	AGTCGAAGACAGATCCGNNKGAT**NNK**ACCTATACCCGGAATATGTACATTG
OppA_L530X_N532X_rev	AGTCGAAGACGCGGATCTTTGCCGGTATAG
OppA_genomic_fwd	TACACATGCTGGTTAATACCAGTAATTATAATGAGGGAGTCCAAAAAACAATGACCAACATCACCAAGAGAAG
OppA_genomic_rev	AAAATCAGACACCGTGGAGCAGGACACTCCTGCCCCACGTATTGCCATTACTTCACAATGTACATATTCCGGG

X01: trimer primers with 20 codons encoding 20 amino acids

Table 3: Primers used for SLIM cloning

Construct	Primer	Sequence
pEVOL_*Mb*PylRS_*opp*A_D445A	Short fwd	TACAACGAACCAACTTCC
	Short rev	ACGGGCCACATCAAAAGTAC
	Tail fwd	GCAGGCTGGTGTGCTGCTTACAACGAACCAACTTCC
	Tail rev	AGCAGCACACCAGCCTGCACG
pEVOL_*Mb*PylRS_*opp*A_R439A	Short fwd	GCAGGCTGGTGTGCTGAC
	Short rev	CTGGTGACGGGTGTCGAG
	Tail fwd	GGTACTTTTGATGTGGCCGCGGCAGGCTGGTGTGCTG
	Tail rev	CGCGGCCACATCAAAAGTACCCTGG

Table 4: Peptides used in this study

Peptide	Expected mass	[M+H]+
G-AisoK(1)	274.32	275.2
AisoK	217.25	218.2
G-SisoK	290.32	291.1
SisoK	233.25	234.2
G-TisoK	304.35	305.2
TisoK	247.28	248.2
G-CisoK	306.38	307.2
CisoK	249.31	250.2
G-VisoK	302.38	303.2
VisoK	245.31	246.2
G-LisoK	316.4	317.2
LisoK	259.33	260.2
G-PisoK	300.36	301.2
PisoK	243.29	244.2
G-HisoK	340.38	341.2
HisoK	283.31	284.2
G-PrgisoK	298.34	299.1
PrgisoK	241.27	242.2
G-pLisoK	328.37	329.2
pLisoK	271.3	272.2
G-ClAisoK	308.76	309.1

ClAisoK	251.7	252.1
K-AisoK (2)	346.45	346.2
AcK-AisoK (3)	388.49	388.2
CbzK-AisoK (4)	480.59	480.3
ONBK-AisoK (5)	525.58	525.3
CouK-AisoK (6)	641.54	642.2
F-AisoK (7)	365.45	365.2
3PyA-AisoK (8)	366.44	366.2
BoF-AisoK (9	409.27	409.2
3mH-AisoK (10)	369.45	369.2
AllocDAP-AisoK (11)	388.44	388.2
BioK-AisoK (12)	572.75	572.3
LipK-AisoK (13)	534.75	534.3
SucK-AisoK (14)	446.52	446.2
GluK-AisoK (15)	460.55	460.3
AcK-pLisoK (16)	441.53	442.3

Table 5 Crystallographic data collection and refinement statistics.

	OppA:G-SisoK
**Data collection**	
Space group	$P2_1$
Cell dimensions	
a, *b*, *c* (Å)	60.0, 200.7, 103.4
α, β, γ (°)	90 95.7 90
Resolution (Å)[a]	30 - 2.5 (2.6 - 2.5)
R_{merge}[a,b,c]	11.9 (68.8)
$I / \sigma I$[a]	7.5 (2.0)
Completeness (%)[a]	95.8 (99.6)
Redundancy[a]	3.0 (3.1)
**Refinement**	
Resolution (Å)	30 - 2.5
No. reflections	80170
R_{work} / R_{free}[d]	0.229 / 0.259
No. atoms	16810
Protein	16617
Ligand/ion	80
Water	113
B-factors	
Protein	36.7
Ligand/ion	36.8
Water	30.2
R.m.s. deviations	
Bond lengths (Å)	0.002
Bond angles (°)	1.19

[a] The values in parentheses for resolution range, completeness, R_{merge} and I/σ (I) correspond to the highest resolution shell

[b] Data reduction was carried out from a single crystal. Friedel pairs were treated as identical reflections

[c] $R_{merge}(I) = \Sigma_{hkl}\Sigma_j \mid I(hkl)_j - \langle I(hkl)\rangle \mid / \Sigma_{hkl} \Sigma_j I(hkl)_j$, where $I(hkl)_j$ is the jth measurement of the intensity of reflection hkl and <I(hkl)> is the average intensity

[d] $R = \Sigma_{hkl} \mid |F_{obs}| - |F_{calc}| \mid / \Sigma_{hkl} |F_{obs}|$, where R_{free} is calculated without a sigma cut off for a randomly chosen 5% of reflections, which were not used for structure refinement, and R_{work} is calculated for the remaining reflections.

Table 6: Mutations in OppA variants

OppA variant	Mutations
Error-prone Variant 1	T173N, **D221**G, K371E, **R439**H
Error-prone Variant 2	L78I, **W222**R, K307R, K333N
Error-prone Variant 3	T429A, **R439**L, **S460**C, V482A
Error-prone Variant 4	V193A, I303V, N337D, **S460**N
Site-saturation variant OppA-iso	V193A, **D221**L, **W222**A, I303V, N337D, **R439**Q, **S460**H
OppA-Z1	**V60**E, **S63**G, **L530**G, **N532**S
OppA-Z2	**S63**A, R439H, **L530**G, **N532**A

In **bold**: positions targeted for site saturation mutagenesis

Table 7: gRNA sequences used in this study:

Targeting site	sgRNA sequence
FRT site in OppA locus of Δ*opp*A cells	TAGAGAATAGGAACTTCGAACTG
pepA	TCCGGGCTACCGCTTTTTACACT,<br>CTTTACCAGAACGCCAGGCGGTA
pepB	AACGGGGCAGACGATCTGGGGCT,<br>TTGAGAACTATCAGCAAGGCTGG
pepT	ATTGGGTATCCAGAGACACGTAG,<br>CATACCTTCCAGAGTCACAAACT
ypdEF	CTTGACGCGACGAACACGCTTAC,<br>CAGATGCAGCAACTTTTATCTGC

Protein Sequences

sfGFP N150TAG H6

MPSKGEELFTGVVPILVELDGDVNGHKFSVRGEGEGDATNGKLTLKFICTTGKLPVP
WPTLVTTLTYGVQCFSRYPDHMKRHDFFKSAMPEGYVQERTISFKDDGTYKTRAEV
KFEGDTLVNRIELKGIDFKEDGNILGHKLEYNFNSH*VYITADKQKNGIKANFKIRHN
VEDGSVQLADHYQQNTPIGDGPVLLPDNHYLSTQSVLSKDPNEKRDHMVLLEFVTA
AGITHGMDELYKGSHHHHHH

UbK63TAG H6

MQIFVKTLTGKTITLEVEPSDTIENVKAKIQDKEGIPPDQQRLIFAGKQLEDGRTLSDY
NIQ*ESTLHLVLRLRGGHHHHHH

***Mb*PylRS**

MDKKPLDVLISATGLWMSRTGTLHKIKHHEVSRSKIYIEMACGDHLVVNNSRSCRT
ARAFRHHKYRKTCKRCRVSDEDINNFLTRSTESKNSVKVRVVSAPKVKKAMPKSVS
RAPKPLENSVSAKASTNTSRSVPSPAKSTPNSSVPASAPAPSLTRSQLDRVEALLSPED
KISLNMAKPFRELEPELVTRRKNDFQRLYTNDREDYLGKLERDITKFFVDRGFLEIKS
PILIPAEYVERMGINNDTELSKQIFRVDKNLCLRPMLAPTLYNYLRKLDRILPGPIKIFE
VGPCYRKESDGKEHLEEFTMVNFCQMGSGCTRENLEALIKEFLDYLEIDFEIVGDSC
MVYGDTLDIMHGDLELSSAVVGPVSLDREWGIDKPWIGAGFGLERLLKVMHGFKNI
KRASRSESYYNGISTNL

***Ma*PylRS H227I Y228P**

MTVKYTDAQIQRLREYGNGTYEQKVFEDLASRDAAFSKEMSVASTDNEKKIKGMIA
NPSRHGLTQLMNDIADALVAEGFIEVRTPIFISKDALARMTITEDKPLFKQVFWIDEK
RALRPMLAPNLYSVMRDLRDHTDGPVKIFEMGSCFRKESHSGMHLEEFTMLNLVD
MGPRGDATEVLKNYISVVMKAAGLPDYDLVQEESDVYKETIDVEINGQEVCSAAVG
PIPLDAAHDVHEPWSGAGFGLERLLTIREKYSTVKKGGASISYLNGAKIN

DiazKRS

MDKKPLNTLISATGLWMSRTGTIHKIKHHEVSRSKIYIEMACGDHLVVNNSRSSRTA
RALRHHKYRKTCKRCRVSDEDLNKFLTKANEDQTSVKVKVVSAPTRTKKAMPKSV
ARAPKPLENTEAAQAQPSGSKFSPAIPVSTQESVSVPASVSTSISSISTGATASALVKG
NTNPITSMSAPVQASAPALTKSQTDRLEVLLNPKDEISLNSGKPFRELESELLSRRKKD
LQQIYAEERENYLGKLEREITRFFVDRGFLEIKSPILIPLEYIERMGIDNDTELSKQIFRV
DKNFCLRPMLAPNLMNYARKLDRALPDPIKIFEIGPCYRKESDGKEHLEEFTMLNFA
QMGSGCTRENLESIITDFLNHLGIDFKIVGDSCMVYGDTLDVMHGDLELSSAVVGPIP
LDREWGIDKPWIGAGFGLERLLKVKHDFKNIKRAARSESYYNGISTNL

eGFP-NB wt

MKYLLPTAAAGLLLLAAQPAMAQVQLVESGGALVQPGGSLRLSCAASGFPVNRYS
MRWYRQAPGKEREWVAGMSSAGDRSSYEDSVKGRFTISRDDARNTVYLQMNSLKP
EDTAVYYCNVNVGFEYWGQGTQVTVSSKKKHHHHHH

eGFP-NB R17TAG H6

MKYLLPTAAAGLLLLAAQPAMAQVQLVESGGALVQPGGSL*LSCAASGFPVNRYS
MRWYRQAPGKEREWVAGMSSAGDRSSYEDSVKGRFTISRDDARNTVYLQMNSLKP
EDTAVYYCNVNVGFEYWGQGTQVTVSSKKKHHHHHH

eGFP-NB R75TAG H6
MKYLLPTAAAGLLLLAAQPAMAQVQLVESGGALVQPGGSLRLSCAASGFPVNRYS
MRWYRQAPGKEREWVAGMSSAGDRSSYEDSVKGRFTISRDDA*NTVYLQMNSLKP
EDTAVYYCNVNVGFEYWGQGTQVTVSSKKKHHHHHH

GST E51TAG H6
MSPILGYWKIKGLVQPTRLLLEYLEEKYEEHLYERDEGDKWRNKKFELGL*FPNLPY
YIDGDVKLTQSMAIIRYIADKHNMLGGCPKERAEISMLEGAVLDIRYGVSRIAYSKDF
ETLKVDFLSKLPEMLKMFEDRLCHKTYLNGDHVTHPDFMLYDALDVVLYMDPMCL
DAFPKLVCFKKRIEAIPQIDKYLKSSKYIAWPLQGWQATFGGGDHPPKHHHHHH

3C-Ub-K63TAG H6
MENLLEVLFQGPGGGGSMQIFVKTLTGKTITLEVEPSDTIENVKAKIQDKEGIPPDQQ
RLIFAGKQLEDGRTLSDYNIQ*ESTLHLVLRLRGGHHHHHH

OppA H6
MGTNITKRSLVAAGVLAALMAGNVALAADVPAGVTLAEKQTLVRNNGSEVQSLDP
HKIEGVPESNISRDLFEGLLVSDLDGHPAPGVAESWDNKDAKVWTFHLRKDAKWSD
GTPVTAQDFVYSWQRSVDPNTASPYASYLQYGHIAGIDEILEGKKPITDLGVKAIDD
HTLEVTLSEPVPYFYKLLVHPSTSPVPKAAIEKFGEKWTQPGNIVTNGAYTLKDWVV
NERIVLERSPTYWNNAKTVINQVTYLPIASEVTDVNRYRSGEIDMTNNSMPIELFQKL
KKEIPDEVHVDPYLCTYYYEINNQKPPFNDVRVRTALKLGMDRDIIVNKVKAQGNM
PAYGYTPPYTDGAKLTQPEWFGWSQEKRNEEAKKLLAEAGYTADKPLTINLLYNTS
DLHKKLAIAASSLWKKNIGVNVKLVNQEWKTFLDTRHQGTFDVARAGWCADYNEP
TSFLNTMLSNSSMNTAHYKSPAFDSIMAETLKVTDEAQRTALYTKAEQQLDKDSAIV
PVYYYVNARLVKPWVGGYTGKDPLDNTYTRNMYIVKHHHHHH

OppA-iso H6
MGTNITKRSLVAAGVLAALMAGNVALAADVPAGVTLAEKQTLVRNNGSEVQSLDP
HKIEGVPESNISRDLFEGLLVSDLDGHPAPGVAESWDNKDAKVWTFHLRKDAKWSD
GTPVTAQDFVYSWQRSVDPNTASPYASYLQYGHIAGIDEILEGKKPITDLGVKAIDD
HTLEVTLSEPVPYFYKLLVHPSTSPAPKAAIEKFGEKWTQPGNIVTNGAYTLKLAVV
NERIVLERSPTYWNNAKTVINQVTYLPIASEVTDVNRYRSGEIDMTNNSMPIELFQKL
KKEIPDEVHVDPYLCTYYYEVNNQKPPFNDVRVRTALKLGMDRDIIVNKVKAQGD
MPAYGYTPPYTDGAKLTQPEWFGWSQEKRNEEAKKLLAEAGYTADKPLTINLLYNT
SDLHKKLAIAASSLWKKNIGVNVKLVNQEWKTFLDTRHQGTFDVAQAGWCADYNE
PTSFLNTMLSNHSMNTAHYKSPAFDSIMAETLKVTDEAQRTALYTKAEQQLDKDSAI
VPVYYYYVNARLVKPWVGGYTGKDPLDNTYTRNMYIVKHHHHHH

OppA Z1
MTNITKRSLVAAGVLAALMAGNVALAADVPAGVTLAEKQTLVRNNGSEVQSLDPH
KIEGEPEGNISRDLFEGLLVSDLDGHPAPGVAESWDNKDAKVWTFHLRKDAKWSDG
TPVTAQDFVYSWQRSVDPNTASPYASYLQYGHIAGIDEILEGKKPITDLGVKAIDDHT
LEVTLSEPVPYFYKLLVHPSTSPVPKAAIEKFGEKWTQPGNIVTNGAYTLKDWVVNE
RIVLERSPTYWNNAKTVINQVTYLPIASEVTDVNRYRSGEIDMTNNSMPIELFQKLKK
EIPDEVHVDPYLCTYYYEINNQKPPFNDVRVRTALKLGMDRDIIVNKVKAQGNMPA
YGYTPPYTDGAKLTQPEWFGWSQEKRNEEAKKLLAEAGYTADKPLTINLLYNTSDL
HKKLAIAASSLWKKNIGVNVKLVNQEWKTFLDTRHQGTFDVARAGWCADYNEPTS
FLNTMLSNSSMNTAHYKSPAFDSIMAETLKVTDEAQRTALYTKAEQQLDKDSAIVP
VYYYVNARLVKPWVGGYTGKDPGDSTYTRNMYIVKH

OppA Z2
MTNITKRSLVAAGVLAALMAGNVALAADVPAGVTLAEKQTLVRNNGSEVQSLDPH
KIEGVPEANISRDLFEGLLVSDLDGHPAPGVAESWDNKDAKVWTFHLRKDAKWSD
GTPVTAQDFVYSWQRSVDPNTASPYASYLQYGHIAGIDEILEGKKPITDLGVKAIDD
HTLEVTLSEPVPYFYKLLVHPSTSPVPKAAIEKFGEKWTQPGNIVTNGAYTLKDWVV
NERIVLERSPTYWNNAKTVINQVTYLPIASEVTDVNRYRSGEIDMTNNSMPIELFQKL
KKEIPDEVHVDPYLCTYYYEINNQKPPFNDVRVRTALKLGMDRDIIVNKVKAQGNM
PAYGYTPPYTDGAKLTQPEWFGWSQEKRNEEAKKLLAEAGYTADKPLTINLLYNTS
DLHKKLAIAASSLWKKNIGVNVKLVNQEWKTFLDTRHQGTFDVAHAGWCADYNEP
TSFLNTMLSNSSMNTAHYKSPAFDSIMAETLKVTDEAQRTALYTKAEQQLDKDSAIV
PVYYYVNARLVKPWVGGYTGKDPGDATYTRNMYIVKH

β-lactamase wt H6
MGADLADRFAELERRYDARLGVYVPATGTTAAIEYRADERFAFCSTFKAPLVAAVL
HQNPLTHLDKLITYTSDDIRSISPVAQQHVQTGMTIGQLCDAAIRYSDGTAANLLLAD
LGGPGGGTAAFTGYLRSLGDTVSRLDAEEPELNRDPPGDERDTTTPHAIALVLQQLV
LGNALPPDKRALLTDWMARNTTGAKRIRAGFPADWKVIDKTGTGDYGRANDIAVV
WSPTGVPYVVAVMSDRAGGGYDAEPREALLAEAATCVAGVLAGSGGSGHHHHHH

β-lactamase K221TAG H6
MGADLADRFAELERRYDARLGVYVPATGTTAAIEYRADERFAFCSTFKAPLVAAVL
HQNPLTHLDKLITYTSDDIRSISPVAQQHVQTGMTIGQLCDAAIRYSDGTAANLLLAD
LGGPGGGTAAFTGYLRSLGDTVSRLDAEEPELNRDPPGDERDTTTPHAIALVLQQLV
LGNALPPDKRALLTDWMARNTTGA*RIRAGFPADWKVIDKTGTGDYGRANDIAVV
WSPTGVPYVVAVMSDRAGGGYDAEPREALLAEAATCVAGVLAGSGGSGHHHHHH

SUMO2 wt H6
MADEKPKEGVKTENNDHINLKVAGQDGSVVQFKIKRHTPLSKLMKAYCERQGLSM
RQIRFRFDGQPINETDTPAQLEMEDEDTIDVFQQQTGGHHHHHH

SUMO2 K45TAG H6
MADEKPKEGVKTENNDHINLKVAGQDGSVVQFKIKRHTPLSKLM*AYCERQGLSM
RQIRFRFDGQPINETDTPAQLEMEDEDTIDVFQQQTGGHHHHHH

Calmodulin wt strep
MADQLTEEQIAEFKEAFSLFDKDGDGTITTKELGTVMRSLGQNPTEAELQDMINEVD
ADGNGTIDFPEFLTMMARKMKDTDSEEEIREAFRVFDKDGNGYISAAELRHVMTNL
GEKLTDEEVDEMIREADIDGDGQVNYEEFVQMMTAKSAWSHPQFEKGGGSGGGSG
GSAWSHPQFEK

Calmodulin G40TAG strep
MADQLTEEQIAEFKEAFSLFDKDGDGTITTKELGTVMRSL*QNPTEAELQDMINEVD
ADGNGTIDFPEFLTMMARKMKDTDSEEEIREAFRVFDKDGNGYISAAELRHVMTNL
GEKLTDEEVDEMIREADIDGDGQVNYEEFVQMMTAKSAWSHPQFEKGGGSGGGSG
GSAWSHPQFEK

Histone H3 wt H6
MARTKQTARKSTGGKAPRKQLATKAARKSAPATGGVKKPHRYRPGTVALREIRRY
QKSTELLIRKLPFQRLVREIAQDFKTDLRFQSSAVMALQEASEAYLVALFEDTNLCAI
HAKRVTIMPKDIQLARRIRGERARSHHHHHH

Histone H3 K112TAG H6
MARTKQTARKSTGGKAPRKQLATKAARKSAPATGGVKKPHRYRPGTVALREIRRY
QKSTELLIRKLPFQRLVREIAQDFKTDLRFQSSAVMALQEASEAYLVALFEDTNLCAI
HAKRVTIMP*DIQLARRIRGERARSHHHHHH

Histone H3 K79 K112TAG H6
MARTKQTARKSTGGKAPRKQLATKAARKSAPATGGVKKPHRYRPGTVALREIRRY
QKSTELLIRKLPFQRLVREIAQDF*TDLRFQSSAVMALQEASEAYLVALFEDTNLCAI
HAKRVTIMP*DIQLARRIRGERARSHHHHHH

Histone H3 K27 K79 K112TAG H6
MARTKQTARKSTGGKAPRKQLATKAAR*SAPATGGVKKPHRYRPGTVALREIRRYQ
KSTELLIRKLPFQRLVREIAQDF*TDLRFQSSAVMALQEASEAYLVALFEDTNLCAIH
AKRVTIMP*DIQLARRIRGERARSHHHHHH

Tyrosinase H6
MSNKYRVRKNVLHLTDTEKRDFVRTVLILKEKGIYDRYIAWHGAAGKFHTPPGSDR
NAAHMSSAFLPWHREYLLRFERDLQSINPEVTLPYWEWETDAQMQDPSQSQIWSAD
FMGGNGNPIKDFIVDTGPFAAGRWTTIDEQGNPSGGLKRNFGATKEAPTLPTRDDVL
NALKITQYDTPPWDMTSQNSFRNQLEGFINGPQLHNRVHRWVGGQMGVVPTAPND
PVFFLHHANVDRIWAVWQIIHRNQNYQPMKNGPFGQNFRDPMYPWNTTPEDVMNH
RKLGYVVYDIELRKSKRSSLEHHHHHH

Hsp82 H7
MASETFEFQAEITQLMSLIINTVYSNKEIFLRELISNASDALDKIRYKSLSDPKQLETEP
DLFIRITPKPEQKVLEIRDSGIGMTKAELINNLGTIAKSGTKAFMEALSAGADVSMIGQ
FGVGFYSLFLVADRVQVISKSNDDEQYIWESNAGGSFTVTLDEVNERIGRGTILRLFL
KDDQLEYLEEKRIKEVIKRHSEFVAYPIQLVVTKEVEKEVPIPEEEKKDEEKKDEEKK
DEDDKKPKLEEVDEEEEKKPKTKKVKEEVQEIEELNKTKPLWTRNPSDITQEEYNAF
YKSISNDWEDPLYVKHFSVEGQLEFRAILFIPKRAPFDLFESKKKKNNIKLYVRRVFIT
DEAEDLIPEWLSFVKGVVDSEDLPLNLSREMLQQNKIMKVIRKNIVKKLIEAFNEIAE
DSEQFEKFYSAFSKNIKLGVHEDTQNRAALAKLLRYNSTKSVDELTSLTDYVTRMPE
HQKNIYYITGESLKAVEKSPFLDALKAKNFEVLFLTDPIDEYAFTQLKEFEGKTLVDIT
KDFELEETDEEKAEREKEIKEYEPLTKALKEILGDQVEKVVVSYKLLDAPAAIRTGQF
GWSANMERIMKAQALRDSSMSSYMSSKKTFEISPKSPIIKELKKRVDEGGAQDKTVK
DLTKLLYETALLTSGFSLDEPTSFASRINRLISLGLNIDEDEETETAPEASTAAPVEEVP
ADTEMEEVDPGEQKCEEWKRRYEKEKEKNARLKGKVEKLEIELARWRPGSAWSHH
HHHHH

Hsp82 D452TAG H7
MASETFEFQAEITQLMSLIINTVYSNKEIFLRELISNASDALDKIRYKSLSDPKQLETEP
CLFIRITPKPEQKVLEIRDSGIGMTKAELINNLGTIAKSGTKAFMEALSAGADVSMIGQ
FGVGFYSLFLVADRVQVISKSNDDEQYIWESNAGGSFTVTLDEVNERIGRGTILRLFL
KDDQLEYLEEKRIKEVIKRHSEFVAYPIQLVVTKEVEKEVPIPEEEKKDEEKKDEEKK
DEDDKKPKLEEVDEEEEKKPKTKKVKEEVQEIEELNKTKPLWTRNPSDITQEEYNAF
YKSISNDWEDPLYVKHFSVEGQLEFRAILFIPKRAPFDLFESKKKKNNIKLYVRRVFIT

DEAEDLIPEWLSFVKGVVDSEDLPLNLSREMLQQNKIMKVIRKNIVKKLIEAFNEIAE
DSEQFEKFYSAFSKNIKLGVHEDTQNRAALAKLLRYNSTKSV*ELTSLTDYVTRMPE
HQKNIYYITGESLKAVEKSPFLDALKAKNFEVLFLTDPIDEYAFTQLKEFEGKTLVDIT
KDFELEETDEEKAEREKEIKEYEPLTKALKEILGDQVEKVVVSYKLLDAPAAIRTGQF
GWSANMERIMKAQALRDSSMSSYMSSKKTFEISPKSPIIKELKKRVDEGGAQDKTVK
DLTKLLYETALLTSGFSLDEPTSFASRINRLISLGLNIDEDEETETAPEASTAAPVEEVP
ADTEMEEVDPGEQKCEEWKRRYEKEKEKNARLKGKVEKLEIELARWRPGSA
WSHHHHHHH

IL-2 wt H6
MAPTSSSTKKTQLQLEHLLLDLQMILNGINNYKNPKLTRMLTFKFYMPKKATELKHL
QCLEEELKPLEEVLNLAQSKNFHLRPRDLISNINVIVLELKGSETTFMCEYADETATIV
EFLNRWITFCQSIISTLTGSGSHHHHHH

IL-2 R38TAG
MAPTSSSTKKTQLQLEHLLLDLQMILNGINNYKNPKLT*MLTFKFYMPKKATELKHL
QCLEEELKPLEEVLNLAQSKNFHLRPRDLISNINVIVLELKGSETTFMCEYADETATIV
EFLNRWITFCQSIISTLTGSGSHHHHHH

TRX H6 hGH wt
MSDKIIHLTDDSFDTDVLKADGAILVDFWAEWCGPCKMIAPILDEIADEYQGKLTVA
KLNIDQNPGTAPKYGIRGIPTLLLFKNGEVAATKVGALSKGQLKEFLDANLAGSGSG
HHHHHHHGSGSGENLYFQGFPTIPLSRLFDNAMLRADRLNQLAFDTYQEFEEAYIPK
EQKYSFLQNPQTSLCFSESIPTPSNREETQQKSNLELLRISLLLIQSWLEPVQFLRSVFA
NSLVYGASDSNVYDLLKDLEEKIQTLMGRLEDGSPRTGQIFKQTYSKFDTNSHNDDA
LLKNYGLLYCFNADMSRVSTFLRTVQCRSVEGSCGF

TRX H6 hGH Y35TAG
MSDKIIHLTDDSFDTDVLKADGAILVDFWAEWCGPCKMIAPILDEIADEYQGKLTVA
KLNIDQNPGTAPKYGIRGIPTLLLFKNGEVAATKVGALSKGQLKEFLDANLAGSGSG
HHHHHHHGSGSGENLYFQGFPTIPLSRLFDNAMLRADRLNQLAFDTYQEFEEA*IPK
EQKYSFLQNPQTSLCFSESIPTPSNREETQQKSNLELLRISLLLIQSWLEPVQFLRSVFA
NSLVYGASDSNVYDLLKDLEEKIQTLMGRLEDGSPRTGQIFKQTYSKFDTNSHNDDA
LLKNYGLLYCFNADMSRVSTFLRTVQCRSVEGSCGF

TRX H6 hGH K38TAG
MSDKIIHLTDDSFDTDVLKADGAILVDFWAEWCGPCKMIAPILDEIADEYQGKLTVA
KLNIDQNPGTAPKYGIRGIPTLLLFKNGEVAATKVGALSKGQLKEFLDANLAGSGSG
HHHHHHHGSGSGENLYFQGFPTIPLSRLFDNAMLRADRLNQLAFDTYQEFEEAYIP*
EQKYSFLQNPQTSLCFSESIPTPSNREETQQKSNLELLRISLLLIQSWLEPVQFLRSVFA
NSLVYGASDSNVYDLLKDLEEKIQTLMGRLEDGSPRTGQIFKQTYSKFDTNSHNDDA
LLKNYGLLYCFNADMSRVSTFLRTVQCRSVEGSCGF

GST TEV RanGAP1 H6 wt
MSPILGYWKIKGLVQPTRLLLEYLEEKYEEHLYERDEGDKWRNKKFELGLEFPNLPY
YIDGDVKLTQSMAIIRYIADKHNMLGGCPKERAEISMLEGAVLDIRYGVSRIAYSKDF
ETLKVDFLSKLPEMLKMFEDRLCHKTYLNGDHVTHPDFMLYDALDVVLYMDPMCL
DAFPKLVCFKKRIEAIPQIDKYLKSSKYIAWPLQGWQATFGGGDHPPKGIEENLYFQG
NTGEPAPVLSSPPPADVSTFLAFPSPEKLLRLGPKSSVLIAQQTDTSDPEKVVSAFLKV
SSVFKDEATVRMAVQDAVDALMQKAFNSSSFNSNTFLTRLLVHMGLLKSEDKVKAI

ANLYGPLMALNHMVQQDYFPKALAPLLLAFVTKPNSALESCSFARHSLLQTLYKVH
HHHHH

GST TEV RanGAP1 H6 K524TAG
MSPILGYWKIKGLVQPTRLLLEYLEEKYEEHLYERDEGDKWRNKKFELGLEFPNLPY
YIDGDVKLTQSMAIIRYIADKHNMLGGCPKERAEISMLEGAVLDIRYGVSRIAYSKDF
ETLKVDFLSKLPEMLKMFEDRLCHKTYLNGDHVTHPDFMLYDALDVVLYMDPMCL
DAFPKLVCFKKRIEAIPQIDKYLKSSKYIAWPLQGWQATFGGGDHPPKGIEENLYFQG
NTGEPAPVLSSPPPADVSTFLAFPSPEKLLRLGPKSSVLIAQQTDTSDPEKVVSAFLKV
SSVFKDEATVRMAVQDAVDALMQKAFNSSSFNSNTFLTRLLVHMGLL*SEDKVKAI
ANLYGPLMALNHMVQQDYFPKALAPLLLAFVTKPNSALESCSFARHSLLQTLYKVH
HHHHH

AcKRS3
MDKKPLDVLISATGLWMSRTGTLHKIKHHEVSRSKIYIEMACGDHLVVNNSRSCRT
ARAFRHHKYRKTCKRCRVSGEDINNFLTRSTESKNSVKVRVVSAPKVKKAMPKSVS
RAPKPLENSVGAKASTNTSRSVPSPAKSTPNSSVPASAPAPSLTRSQLDRVEALLSPED
KISLNMAKPFRELEPELVTRRKNDFQRLYTNDREDYLGKLERDITKFFVDRGFLEIKS
PILIPAEYVERMGINNDTELSKQIFRVDKNLCLRPMMAPTIFNYARKLDRILPGPIKIFE
VGPCYRKESDGKEHLEEFTMVNFFQMGSGCTRENLEALIKEFLDYLEIDFEIVGDSC
MVYGDTLDIMHGDLELSSAVVGPVSLDREWGIDKPWIGAGFGLERLLKVMHGFKNI
KRASRSESYYNGISTNL

***Mm*PylRS Y306A Y384A**
MDKKPLNTLISATGLWMSRTGTIHKIKHHEVSRSKIYIEMACGDHLVVNNSRSSRTA
RALRHHKYRKTCKRCRVSDEDLNKFLTKANEDQTSVKVKVVSAPTRTKKAMPKSV
ARAPKPLENTEAAQAQPSGSKFSPAIPVSTQESVSVPASVSTSISSISTGATASALVKG
NTNPITSMSAPVQASAPALTKSQTDRLEVLLNPKDEISLNSGKPFRELESELLSRRKKD
LQQIYAEERENYLGKLEREITRFFVDRGFLEIKSPILIPLEYIERMGIDNDTELSKQIFRV
DKNFCLRPMLAPNLANYLRKLDRALPDPIKIFEIGPCYRKESDGKEHLEEFTMLNFCQ
MGSGCTRENLESIITDFLNHLGIDFKIVGDSCMVFGDTLDVMHGDLELSSAVVGPIPL
DREWGIDKPWIGAGFGLERLLKVKHDFKNIKRAARSESYYNGISTNL

***Mb*PylRS Y271A L274M**
MDKKPLDVLISATGLWMSRTGTLHKIKHHEVSRSKIYIEMACGDHLVVNNSRSCRT
ARAFRHHKYRKTCKRCRVSDEDINNFLTRSTESKNSVKVRVVSAPKVKKAMPKSVS
RAPKPLENSVSAKASTNTSRSVPSPAKSTPNSSVPASAPAPSLTRSQLDRVEALLSPED
KISLNMAKPFRELEPELVTRRKNDFQRLYTNDREDYLGKLERDITKFFVDRGFLEIKS
PILIPAEYVERMGINNDTELSKQIFRVDKNLCLRPMLAPTLANYMRKLDRILPGPIKIF
EVGPCYRKESDGKEHLEEFTMVNFCQMGSGCTRENLEALIKEFLDYLEIDFEIVGDSC
MVYGDTLDIMHGDLELSSAVVGPVSLDREWGIDKPWIGAGFGLERLLKVMHGFKNI
KRASRSESYYNGISTNL

sfGFP N40TAA N150TAG H6
MPSKGEELFTGVVPILVELDGDVNGHKFSVRGEGEGDAT*GKLTLKFICTTGKLPVP
WPTLVTTLTYGVQCFSRYPDHMKRHDFFKSAMPEGYVQERTISFKDDGTYKTRAEV
KFEGDTLVNRIELKGIDFKEDGNILGHKLEYNFNSH*VYITADKQKNGIKANFKIRHN
VEDGSVQLADHYQQNTPIGDGPVLLPDNHYLSTQSVLSKDPNEKRDHMVLLEFVTA
AGITHGMDELYKGSHHHHHH

Ub K48TAA TEV SUMO2K11TAG H6
MQIFVKTLTGKTITLEVEPSDTIENVKAKIQDKEGIPPDQQRLIFAG*QLEDGRTLSDY
NIQKESTLHLVLRLRGGEDLYFQSMADEKPKEGVKTENNDHINL*VAGQDGSVVQF
KIKRHTPLSKLMKAYCERQGLSMRQIRFRFDGQPINETDTPAQLEMEDEDTIDVFQQ
QQNGLHHHHHH

References

1 Serfling, R. *et al.* Designer tRNAs for efficient incorporation of non-canonical amino acids by the pyrrolysine system in mammalian cells. *Nucleic Acids Res* 46, 1-10 (2018). https://doi.org/10.1093/nar/gkx1156

2 Tai, J. *et al.* Pyrrolysine-Inspired in Cellulo Synthesis of an Unnatural Amino Acid for Facile Macrocyclization of Proteins. *J Am Chem Soc* 145, 10249-10258 (2023). https://doi.org/10.1021/jacs.3c01291

3 Zang, J. *et al.* Genetic code expansion reveals aminoacylated lysine ubiquitination mediated by UBE2W. *Nat Struct Mol Biol* 30, 62-71 (2023). https://doi.org/10.1038/s41594-022-00866-9

4 Nguyen, D. P. *et al.* Genetic Encoding and Labeling of Aliphatic Azides and Alkynes in Recombinant Proteins via a Pyrrolysyl-tRNA Synthetase/tRNACUA Pair and Click Chemistry. *Journal of the American Chemical Society* 131, 8720-8721 (2009). https://doi.org/10.1021/ja900553w

5 Chiu, J., March, P. E., Lee, R. & Tillett, D. Site-directed, Ligase-Independent Mutagenesis (SLIM): a single-tube methodology approaching 100% efficiency in 4 h. *Nucleic Acids Res* 32, e174 (2004). https://doi.org/10.1093/nar/gnh172

6 Kabsch, W. Xds. *Acta Crystallogr D Biol Crystallogr* 66, 125-132 (2010). https://doi.org/10.1107/S0907444909047337

7 McCoy, A. J. *et al.* Phaser crystallographic software. *J Appl Crystallogr* 40, 658-674 (2007). https://doi.org/10.1107/S0021889807021206

8 Klepsch, M. M. *et al.* Escherichia coli peptide binding protein OppA has a preference for positively charged peptides. *J Mol Biol* 414, 75-85 (2011). https://doi.org/10.1016/j.jmb.2011.09.043

9 Murshudov, G. N. *et al.* REFMAC5 for the refinement of macromolecular crystal structures. *Acta Crystallogr D Biol Crystallogr* 67, 355-367 (2011). https://doi.org/10.1107/S0907444911001314

10 Emsley, P., Lohkamp, B., Scott, W. G. & Cowtan, K. Features and development of Coot. *Acta Crystallogr D Biol Crystallogr* 66, 486-501 (2010). https://doi.org/10.1107/S0907444910007493

11 Morris, R. J., Perrakis, A. & Lamzin, V. S. ARP/wARP and automatic interpretation of protein electron density maps. *Methods Enzymol* 374, 229-244 (2003). https://doi.org/10.1016/S0076-6879(03)74011-7

12 Williams, C. J. *et al.* MolProbity: More and better reference data for improved all-atom structure validation. *Protein Sci* 27, 293-315 (2018). https://doi.org/10.1002/pro.3330

13 Muzika, M. *et al.* Chemically-defined lactose-based autoinduction medium for site-specific incorporation of non-canonical amino acids into proteins. *RSC Adv* 8, 25558-25567 (2018). https://doi.org/10.1039/c8ra04359k

14 Nguyen, T. A., Gronauer, T. F., Nast-Kolb, T., Sieber, S. A. & Lang, K. Substrate Profiling of Mitochondrial Caseinolytic Protease P via a Site-Specific Photocrosslinking Approach. *Angew Chem Int Ed Engl* 61, e202111085 (2022). https://doi.org/10.1002/anie.202111085
