# Supplementary material for: Hijacking a bacterial ABC transporter for genetic code expansion
Source: Nature. 2025 Oct 15;647(8091):1045–53. doi: 10.1038/s41586-025-09576-w (PMC12657241; doi:10.1038/s41586-025-09576-w)
Supplement: Supplementary file 2 — Reporting Summary [file 41586_2025_9576_MOESM2_ESM.pdf]

nature portfolio

Corresponding author(s): Kathrin Lang

Last updated by author(s): Jul 31, 2025

Reporting Summary

Nature Portfolio wishes to improve the reproducibility of the work that we publish. This form provides structure for consistency and transparency in reporting. For further information on Nature Portfolio policies, see our Editorial Policies and the Editorial Policy Checklist.

Statistics

For all statistical analyses, confirm that the following items are present in the figure legend, table legend, main text, or Methods section.

n/a	Confirmed	
☐	☒	The exact sample size (*n*) for each experimental group/condition, given as a discrete number and unit of measurement
☐	☒	A statement on whether measurements were taken from distinct samples or whether the same sample was measured repeatedly
☒	☐	The statistical test(s) used AND whether they are one- or two-sided<br>*Only common tests should be described solely by name; describe more complex techniques in the Methods section.*
☒	☐	A description of all covariates tested
☒	☐	A description of any assumptions or corrections, such as tests of normality and adjustment for multiple comparisons
☐	☒	A full description of the statistical parameters including central tendency (e.g. means) or other basic estimates (e.g. regression coefficient) AND variation (e.g. standard deviation) or associated estimates of uncertainty (e.g. confidence intervals)
☒	☐	For null hypothesis testing, the test statistic (e.g. *F*, *t*, *r*) with confidence intervals, effect sizes, degrees of freedom and *P* value noted<br>*Give P values as exact values whenever suitable.*
☒	☐	For Bayesian analysis, information on the choice of priors and Markov chain Monte Carlo settings
☒	☐	For hierarchical and complex designs, identification of the appropriate level for tests and full reporting of outcomes
☒	☐	Estimates of effect sizes (e.g. Cohen's *d*, Pearson's *r*), indicating how they were calculated

Our web collection on statistics for biologists contains articles on many of the points above.

Software and code

Policy information about availability of computer code

Data collection	All gels and western blots were imaged using: iBright Imager TM Smart Digital Imaging (Thermo Fischer Scientific, 1.8.1.), Amersham ImageQuant 800 Control Software (Cytiva, 2.0.0)<br>LC-MS data was collected using OpenLab ChemStation (Agilent, LTS01.11 (251).
Data analysis	Graphpad Prism 10 was used to generate all graphs in this study, data analysis for microscale thermophoresis were performed on MO.affinity Analysis (v3.0.5, NanoTemper Technologies).<br>LC-MS data was analyzed on OpenLab ChemStation (Agilent, LTS01.11 (251)<br>XDS , REFMAC 5, ARP/wARP 8.0, PRODRG, MOLPROBITY 4.0.4, PHASER 2.7.0, COOT v0.9, PyMol 2.3.5 and CCP4 suite 7.0 for X-ray structure analysis

For manuscripts utilizing custom algorithms or software that are central to the research but not yet described in published literature, software must be made available to editors and reviewers. We strongly encourage code deposition in a community repository (e.g. GitHub). See the Nature Portfolio guidelines for submitting code & software for further information.

Data

Policy information about availability of data

All manuscripts must include a data availability statement. This statement should provide the following information, where applicable:

- Accession codes, unique identifiers, or web links for publicly available datasets
- A description of any restrictions on data availability
- For clinical datasets or third party data, please ensure that the statement adheres to our policy

Source data for graphs in this study can be found in supplementary data. Uncropped and unprocessed gels can be found in Supplementary information Figure S20. A list of plasmids (Table 1) oligonucleotides (Table 2, 3) and protein sequences used in this study are available in Supplementary Information. All other relevant data is present in the main text, supplementary information and methods. Any additional data is available upon request from the corresponding author. Crystallographic data for the OppA:GSisoK structure was deposited in the RCSB Protein Data Bank with the PDB identification numbers 9RD1. Other X-ry crystal structures mentioned in the paper are available in the RCSB Protein Data bank under identifications numbers: 3TCF, 1GFL, 1LP1, 3JZA.

Research involving human participants, their data, or biological material

Policy information about studies with human participants or human data. See also policy information about sex, gender (identity/presentation), and sexual orientation and race, ethnicity and racism.

Reporting on sex and gender	not applicabe
Reporting on race, ethnicity, or other socially relevant groupings	not applicable
Population characteristics	not applicable
Recruitment	not applicable
Ethics oversight	not applicable

Note that full information on the approval of the study protocol must also be provided in the manuscript.

Field-specific reporting

Please select the one below that is the best fit for your research. If you are not sure, read the appropriate sections before making your selection.

☒ Life sciences ☐ Behavioural & social sciences ☐ Ecological, evolutionary & environmental sciences

For a reference copy of the document with all sections, see nature.com/documents/nr-reporting-summary-flat.pdf

Life sciences study design

All studies must disclose on these points even when the disclosure is negative.

Sample size	No statistical methods were used to determine sample size. For all experiments (SDS-PAGE, western blot, fluorescnce traces, LC-MS assays as well as determination of binding constants) three distinct replicates were anaylzed which is common practice in biological sciences.
Data exclusions	no data was excluded
Replication	For all experiments three distinct replicates were performed and all attempts at replication were successful.
Randomization	Randomization was not relevant for this study. Potential covariates were controlled by ensuring uniform handling across all samples. All samples were processed using standardized protocols, and exposure to procedural variation was minimized.
Blinding	Blinding was not performed as experimental conditions were evident. All samples were processed using standardized protocols, and exposure to procedural variation was minimized.

Reporting for specific materials, systems and methods

We require information from authors about some types of materials, experimental systems and methods used in many studies. Here, indicate whether each material, system or method listed is relevant to your study. If you are not sure if a list item applies to your research, read the appropriate section before selecting a response.

Materials & experimental systems

n/a	Involved in the study
☐	☒ Antibodies
☒	☐ Eukaryotic cell lines
☒	☐ Palaeontology and archaeology
☒	☐ Animals and other organisms
☒	☐ Clinical data
☒	☐ Dual use research of concern
☒	☐ Plants

Methods

n/a	Involved in the study
☒	☐ ChIP-seq
☒	☐ Flow cytometry
☒	☐ MRI-based neuroimaging

Antibodies

Antibodies used

anti-His-HRP dilution 1:10000 (Anti-His-Preoxidase, Roche Cat. no. 11965085001), Anti-Strep-HRP 1:10000 (StrepMAB-Classic HRP, IBA, Cat.No. 2-1509-001).

Validation

The validation statements of the commercial antibodies are available on the website of the suppliers:

anti-His-HRP (Roche Cat. no. 11965085001): https://www.sigmaaldrich.com/CH/en/product/roche/11965085001
anti-Strep-HRP (Cat.No. 2-1509-001): https://www.iba-lifesciences.com/strepmab-classic-hrp/2-1509-001

Plants

Seed stocks

not applicable

Novel plant genotypes

not applicable

Authentication

not applicable
